# AI-ECG-derived biological age as a predictor of mortality in cardiovascular and acute care patients

**DOI:** 10.1093/ehjdh/ztaf109

**Published:** 2025-10-03

**Authors:** Daniel Pavluk, Fabian Theurl, Samuel Proell, Michael Schreinlecher, Florian Hofer, Patrick Rockenschaub, Angus Nicolson, Mercedes Gauthier, Sebastian Reinstadler, Clemens Dlaska, Axel Bauer

**Affiliations:** University Clinic of Internal Medicine III, Cardiology and Angiology—Medical University Innsbruck, Anichstraße 35, Innsbruck AT-6020, Austria; University Clinic of Internal Medicine III, Cardiology and Angiology—Medical University Innsbruck, Anichstraße 35, Innsbruck AT-6020, Austria; Digital Cardiology Lab, University Clinic of Internal Medicine III—Medical University Innsbruck, Andreas-Hofer-Straße 4, Innsbruck AT-6020, Austria; University Clinic of Internal Medicine III, Cardiology and Angiology—Medical University Innsbruck, Anichstraße 35, Innsbruck AT-6020, Austria; University Clinic of Internal Medicine III, Cardiology and Angiology—Medical University Innsbruck, Anichstraße 35, Innsbruck AT-6020, Austria; Institute of Clinical Epidemiology, Public Health, Health Economics, Medical Statistics and Informatics—Medical University Innsbruck, Austria; Digital Cardiology Lab, University Clinic of Internal Medicine III—Medical University Innsbruck, Andreas-Hofer-Straße 4, Innsbruck AT-6020, Austria; University Clinic of Internal Medicine III, Cardiology and Angiology—Medical University Innsbruck, Anichstraße 35, Innsbruck AT-6020, Austria; University Clinic of Internal Medicine III, Cardiology and Angiology—Medical University Innsbruck, Anichstraße 35, Innsbruck AT-6020, Austria; University Clinic of Internal Medicine III, Cardiology and Angiology—Medical University Innsbruck, Anichstraße 35, Innsbruck AT-6020, Austria; Digital Cardiology Lab, University Clinic of Internal Medicine III—Medical University Innsbruck, Andreas-Hofer-Straße 4, Innsbruck AT-6020, Austria; University Clinic of Internal Medicine III, Cardiology and Angiology—Medical University Innsbruck, Anichstraße 35, Innsbruck AT-6020, Austria

**Keywords:** Artificial intelligence, Electrocardiogram (ECG), Biological age, Cardiovascular disease, Risk stratification, Mortality prediction

## Abstract

**Aims:**

Artificial Intelligence (AI) models applied to standard 12-lead ECGs enable estimation of biological age (AI-ECG age), which has shown prognostic value in general populations. However, its clinical utility in high-risk patients with cardiovascular disease (CVD) or acute medical conditions remains insufficiently explored.

**Methods and results:**

We analysed the first ECG of 48 950 consecutive patients presenting to a tertiary care centre with CVD or acute illness between 2000 and 2021. AI-ECG age was derived using a validated deep learning model. Δ-age, defined as the difference between AI-ECG and chronological age, was analysed categorically (±8 years) and continuously using multivariable Cox models adjusted for clinical and ECG variables. Primary endpoint was long-term total mortality (up to 10 years). Saliency map analysis was performed to identify input regions that the model was most sensitive to. AI-ECG age correlated strongly with chronological age (*r* = 0.72, *P* < 0.001), though this correlation weakened in patients with multiple comorbidities. Patients with a positive Δ-age (≥+8 years) had significantly higher 10 year mortality risk (HR: 1.45, *P* < 0.001), while those with a negative Δ-age (≤−8 years) had lower risk (HR: 0.88, *P* < 0.001). These associations were consistent across care settings and remained robust when Δ-age was analysed continuously. Saliency maps indicated that the AI model was most sensitive to the *P*-wave.

**Conclusion:**

AI-ECG age is a strong and independent predictor of long-term mortality in cardiovascular and acute care patients.

## Graphical Abstract

**Graphical Abstract:**
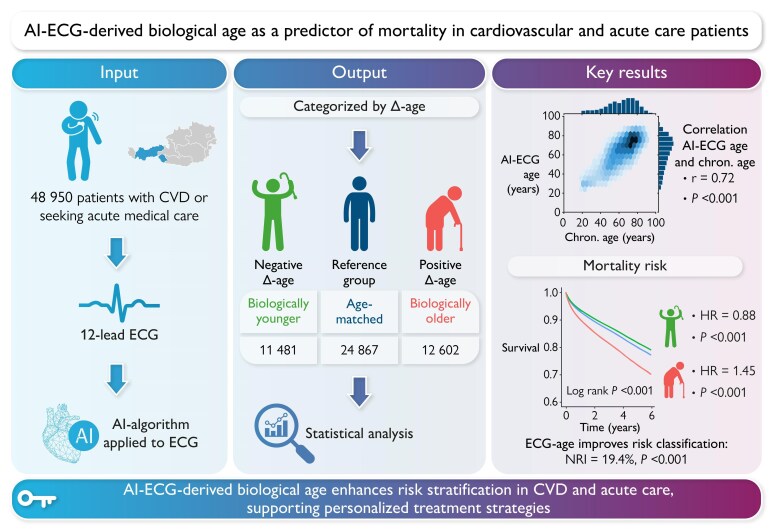

## Introduction

Cardiovascular disease (CVD) remains the leading cause of death and disability-adjusted life years worldwide.^1^ With an ageing global population, its impact is expected to continue to increase.^2,3^ The electrocardiogram (ECG) remains a cornerstone of diagnosis as it is cost-effective, non-invasive, and widely available.^4–6^ Despite its utility, traditional ECG analysis is often limited by its reliance on predefined signal features, rule-based algorithms, and individual expertise in interpretation.^7,8^

In recent years, advances in artificial intelligence (AI), particularly deep neural networks (DNNs), have provided new opportunities to uncover hidden patterns within ECG signals that are not accessible using conventional methods.^9–12^ Unlike traditional approaches that rely on predefined features, DNNs process raw ECG waveforms to automatically learn relevant features, enabling the identification of subtle patterns associated with ageing, disease, and other physiological or pathophysiological characteristics.^13^ AI approaches have successfully developed accurate models for predicting CVD risk and mortality.^14–16^

A particularly promising application of AI-driven ECG analysis is the estimation of biological age from ECG signals, referred to as AI-ECG age.^17,18^ The difference between AI-ECG age and chronological age, Δ-age, has been shown to be predictive of mortality and cardiovascular outcomes across different study populations. Previous studies investigating AI-ECG age and mortality have primarily relied on data from preventive care clinics, where the prevalence of CVD is relatively low.^19–22^ To date, the relationship between Δ-age and all-cause mortality has not been systematically evaluated in patients with known high-risk CVD, including both outpatients and inpatients, as well as individuals presenting to a medical emergency department (ED).

This gap in evidence raises a critical question: Does AI-ECG age generalize as a prognostic biomarker across higher risk populations? Addressing this question is essential for determining the clinical utility of AI-ECG age beyond population-based risk prediction.

To bridge this gap, this study aims to validate an open-source DNN-based model for predicting AI-ECG age in a hospital-based cardiology population, including outpatients and hospitalized patients with established CVD, as well as patients admitted to a medical ED for acute cardiovascular events or other urgent conditions. By analysing this heterogeneous group of patients, this study seeks to evaluate the prognostic value of AI-ECG age in routine cardiovascular care, with a particular focus on its association with all-cause mortality and its potential role in enhancing risk stratification for patients with CVD.

## Methods

### Data source, population, and follow-up

We analysed data from 74 175 patients with a total of 234 945 ECGs recorded at the Medical University Hospital Innsbruck, Tyrol, Austria, between 2000 and 2021. These patients were seen in the cardiology inpatient and outpatient clinic as well as the ED. Patients younger than 18 years were excluded from the analysis (*n* = 346).

**Figure 1:**
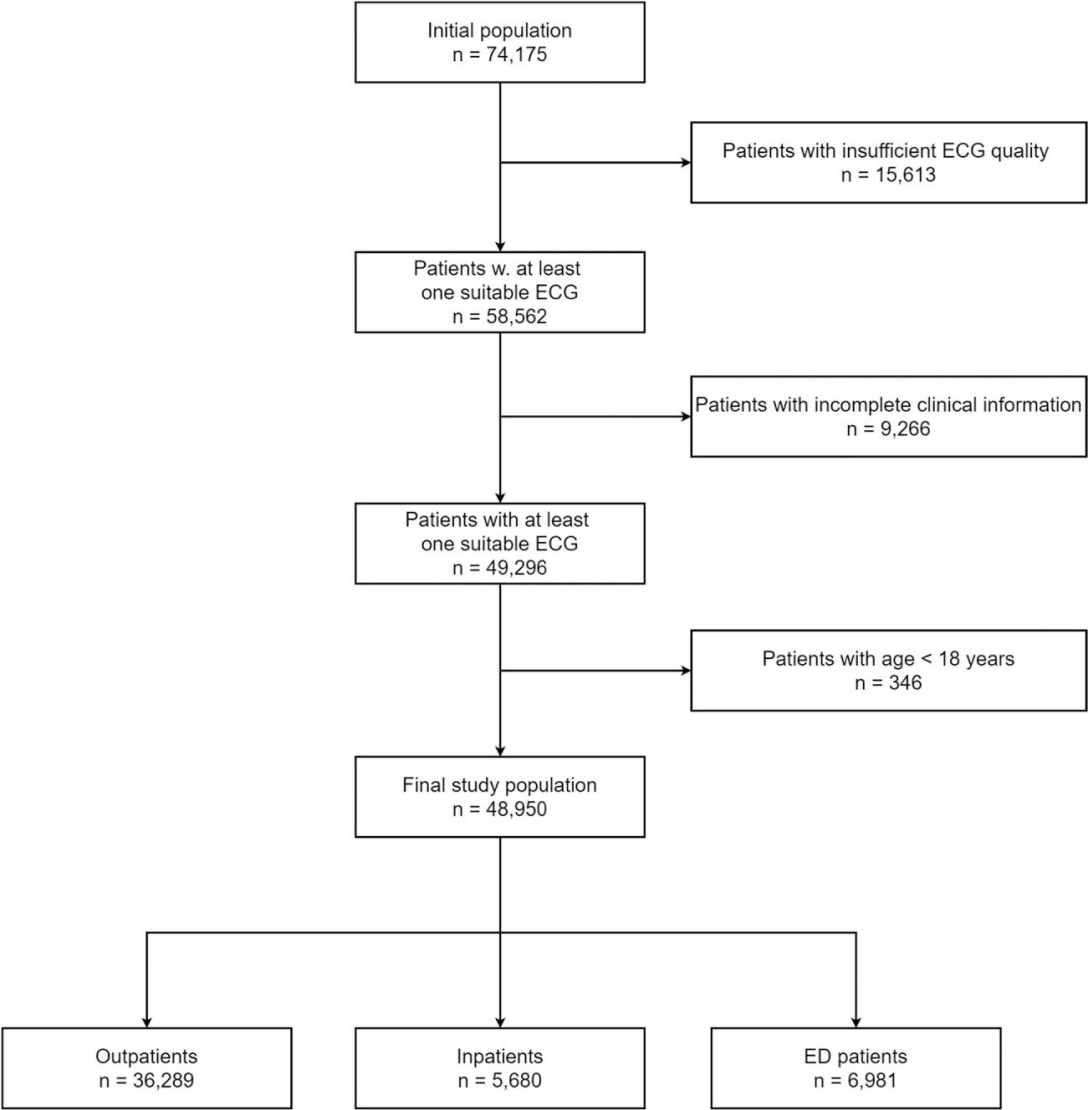
Study population flowchart. A CONSORT-style diagram illustrating the selection process for the final study population and subpopulations.

We retrospectively collected clinical data from hospital registries, resulting in a final dataset of 48 950 patients after excluding 9266 individuals with incomplete clinical information and 15 613 patients due to poor ECG quality (*Figure 1*). Based on patient admission, we defined four cohorts: a total cohort (*n* = 48 950) including all patients and three subcohorts stratified by the clinical setting in which the ECG was recorded: outpatient (*n* = 36 289), inpatient (*n* = 5680), and ED (*n* = 6981) cohorts.

Our dataset included demographic characteristics, medical history, and clinical outcomes, all precisely matched to the time of ECG recording. We used the International Classification of Diseases Tenth Revision (ICD-10) to define specific CVD-related diseases.^23^ We screened the hospital registry for a predefined list of ICD-10 codes to identify relevant diagnoses (see Supplementary material online, *Table S1*) that were present at time of ECG recording as well as that became apparent during follow-up. Future cardiovascular diagnoses were captured via newly documented ICD-10 codes during subsequent hospital visits occurring after the baseline ECG.

The primary endpoint of our study was death of any cause. We retrieved mortality data from the electronic medical record through data access from the federal institution Statistics Austria. For the current analyses, mortality data were last updated on 22 April 2023.

Follow-up information on death was available for all patients (100%). Information on the ICD-10 code was available for 44 674 of the 48 950 patients (91.3%).

Median follow-up was 1167 days (CI: 1144–1189). All follow-up data were collected from the date of ECG recording until 22 April 2023, when clinical information was assessed (see Supplementary material online, *Tables S2* and *S3*).

The Medical University Hospital Innsbruck is a tertiary care centre responsible for managing all major cardiovascular diseases in Tyrol, a region with ∼760 000 inhabitants. As the region’s primary referral centre, it provides specialized care for complex cases. Additionally, it houses the only cardiac catheterization laboratories for acute patients, ensuring access to interventional procedures for time-sensitive conditions.

This study was approved by the Institutional Ethics Committee (No. 1244/2022).

### ECG acquisition

A total of 234 943 ECGs were collected from recordings performed on GE ECG machines (GE HealthCare Technologies; Chicago, USA): MAC2000, MAC5000, MAC5500, and MAC700. Raw ECG data were stored and exported in.XML format for further processing and analysis.

We also extracted automated measurements, including intervals and amplitudes, using the machines’ built-in analysis software. To ensure data quality, we excluded 45 510 ECGs from 15 613 patients due to incorrect lead placement, insufficient recording time, or other technical errors. After filtering out ECGs unsuitable for AI analysis, we retained 189 433 ECGs suitable for further analysis. For patients with multiple ECG recordings, we used the first recorded ECG in our database.

### Data processing and age estimation

Pre-processing of the raw ECG data included extracting signals for each ECG lead, resampling the signal from 500 to 400 Hz, and converting amplitudes from microvolts (µV) to millivolts (mV). These standardized steps ensured consistency in data input and compatibility with the AI algorithm. Pre-processing was performed using Python (version 3.13.1) with the SciPy library.^24^

For age estimation, we used a validated open-source AI algorithm trained on data from 1 558 415 patients to predict a patient’s ‘ECG age’ directly from their ECG signals. The CODE^25^ study cohort was used to develop a DNN based on a residual network architecture to predict patient age from raw ECG tracings.^26^ The development and validation of the AI-ECG age model, including its evaluation using the CODE, ELSA-Brasil, and SaMi-Trop datasets, has been described in detail previously.^22^ The AI model was applied without any recalibration or modification. To ensure comparability with previous studies, we used the originally published version of the model as provided, allowing for direct benchmarking against prior findings.

The AI model used only the pre-processed raw signals as input, without access to patient-specific metadata such as age, gender, or clinical history. No information about the time or date of the ECG recording was provided to the algorithm. The algorithm generates the estimated AI-ECG age as a numerical value with two decimal places.

### Group classification based on the difference between AI-ECG age and chronological age

To assess the prognostic value of AI-ECG age, we divided patients into three groups based on the difference between AI-ECG age and chronological age, referred to as Δ-age.^18^ We divided patients into the following three groups: negative Δ-age, AI-ECG age ≥8 years younger than chronological age; positive Δ-age, AI-ECG age ≥8 years older than chronological age; and reference group, AI-ECG age within ±8 years of chronological age. The chosen ±8 year threshold reflects prior studies^22^ and corresponded to ∼1 SD from the original training error in the AI model. This choice also allowed for an intuitive classification, dividing the population into roughly balanced groups (∼25% negative, 50% reference, 25% positive).

Additionally, we identified prognostically relevant thresholds of Δ-age by systematically varying the cutoff value and calculating the log-rank χ^2^ statistics.

### Explainability of AI-ECG age and explorative analyses

To investigate which parts of the ECG input signal the AI model is most sensitive to, we drew a random subset of 20 000 ECGs from the entire database and computed gradient-based saliency maps for each.^27^ A subset was used to limit the computational load. Saliency maps highlight the regions of the input signal that the model is most sensitive to.

For further analysis, we used automatic ECG delineation to identify on- and offsets of P, QRS and *T*-waves, using neurokit.^28^ Saliency was mean-averaged within each wave as well as within segments between the waves. We computed the mean saliency by identifying R peaks, aligning each heart cycle centred on the R peak, warping cycles to a uniform length, and calculating the average saliency at each time point across cycles.

To assess the temporal stability of Δ-age, we identified patients with repeated ECG recordings spaced at least 1 year apart. We first sorted all available ECGs by patient and recording date, and selected individuals who had at least two ECGs recorded ≥365 days apart. For each of these patients, we extracted all corresponding ECGs and quantified intra-individual Δ-age variability over time. Specifically, we calculated the standard deviation, range, and mean of Δ-age per patient, along with the total number of ECGs and the time span between the earliest and latest ECG. We tested whether the number of hospital admissions during the period between ECG recordings was associated with variability in Δ-age. For this purpose, we used linear regression, modelling the standard deviation of Δ-age as a function of the total number of hospital admissions per patient.

### Statistical analysis

All statistical analyses were conducted using R (version 4.4.1).^29^ Continuous variables were summarized as means with standard deviations or medians with interquartile ranges, as appropriate. Comparisons between two groups were performed using the Mann–Whitney *U* test, while comparisons across more than two groups were conducted using the Kruskal–Wallis test. Categorical variables were expressed as absolute numbers and percentages and compared using two-sided χ^2^ tests or Fisher’s exact test when expected frequencies were low. Statistical significance was defined as *P* < 0.05. Correlations were assessed using Pearson correlation coefficient, while differences between correlation coefficients were analysed with an adaptation of Fisher’s exact test using the CorCor package in R.^30^ Pearson correlation was chosen instead of Spearman correlation because the relationship was approximately linear, as confirmed by visual inspection of scatter plots.

To evaluate the prognostic value of Δ-age, we applied four Cox proportional hazards models. Proportional hazards assumptions for Cox models were tested using Schoenfeld residuals. No significant violations were observed. The first model was adjusted for chronological age and sex. The second model included additional adjustments for arterial hypertension, stroke, coronary artery disease, chronic kidney disease, diabetes mellitus, and atrial fibrillation. The third model included adjustments for PQ interval, *P* duration, QRS duration, QT interval, and RR interval. The fourth model combined all variables from the second and third models. hazard ratios (HRs) with 95% confidence intervals (CIs) were reported for each model. Follow-up times were censored at the last recorded follow-up or death, and for graphical survival analysis, age-adjusted Kaplan–Meier curves were constructed using the ‘adjustedCurves’ package in R.^31^ To further explore the robustness of Δ-age, we conducted subgroup analyses by age categories, <40, 40–60, and >60 years.

To evaluate potential non-linear relationships between Δ-age and mortality, we conducted additional analyses using natural cubic splines with three degrees of freedom within the Cox proportional hazards framework, adjusted for age and gender. This approach allowed us to model potential J-shaped relationships, acknowledging that extreme values of Δ-age (both negative and positive) could carry increased risk compared with moderate deviations. The spline analysis was performed using the R package splines.^32^

To assess the ability of the models to predict mortality, we evaluated their performance using the area under the curve (AUC) of receiver operating characteristic (ROC) curves. We generated time-dependent ROC curves to account for variations in follow-up duration and dynamic risk prediction, using the ‘timeROC’ package in R.^33^ To further quantify the incremental predictive value of incorporating Δ-age, we calculated the net reclassification improvement (NRI) and integrated discrimination improvement (IDI) for all four Cox models using the ‘NRIcens’ package in R.^34^ To account for varying follow-up durations across patient cohorts, we reported separate AUC values for 30 day, 1 year, and 10 year mortality.

Additionally, we explored whether Δ-age could predict new CV diagnoses in the future using two-sided logistic regression, adjusting for age and sex to control for confounders.

## Results

A total of 48 950 patients, each with a single ECG recording, were included in the analysis. *Table 1* shows the baseline characteristics of the total cohort. Baseline characteristics of the subcohorts are available in the supplements (see Supplementary material online, *Tables S4–S6*). Mean age was 62 (± 16.70) years and 45% were female. The most prevalent morbidities were coronary artery disease (37%) and arterial hypertension (39%). The discharge diagnoses in ED patients were most frequently related to cardiology (19%), endocrinology (12%), and allergology/immunology (10%; Supplementary material online, *Table S7*).

**Table 1:** Baseline characteristics for total population *n* = 48 950

	Total *n* = 48 950	Positive Δ-age *n* = 12 602	Reference group *n* = 24 867	Negative Δ-age *n* = 11 481	*P*-value
Chronological age (years)	62.19 (16.73)	52.99 (15.54)	62.90 (16.4)	70.72 (13.5)	<0.001
AI-ECG age (years)	62.65 (16.00)	68.54 (13.90)	63.03 (16.10)	55.36 (13.90)	<0.001
Δ-age (years)^a^	0.46 (12.20)	15.55 (6.83)	0.12 (4.48)	−15.13 (6.33)	<0.001
Females	22 216 (45.39%)	4783 (38.00%)	11 310 (45.50%)	6123 (53.30%)	<0.001
Arterial hypertension	19 031 (38.88%)	4310 (34.20%)	9902 (39.82%)	4819 (41.97%)	<0.001
Diabetes mellitus	5780 (11.81%)	1606 (12.74%)	2971 (11.95%)	1203 (10.48%)	<0.001
Dyslipidaemia	11 804 (24.11%)	2710 (21.50%)	6164 (24.79%)	2930 (25.52%)	<0.001
Coronary artery disease	17 895 (36.56%)	4298 (34.11%)	9239 (37.15%)	4358 (37.96%)	<0.001
Chronic kidney disease	4450 (9.09%)	1123 (8.91%)	2188 (8.80%)	1139 (9.92%)	0.003
Previous myocardial infarction	3970 (8.11%)	1016 (8.06%)	2047 (8.23%)	907 (7.90%)	0.530
Heart failure	2782 (5.68%)	632 (5.02%)	1369 (5.51%)	781 (6.80%)	<0.001
Peripheral arterial disease	3215 (6.57%)	782 (6.21%)	1645 (6.62%)	788 (6.86%)	0.116
Previous stroke	2827 (5.78%)	574 (4.55%)	1453 (5.84%)	800 (6.97%)	0.127
Atrial fibrillation	7910 (16.16%)	1953 (15.50%)	4044 (16.26%)	1913 (16.66%)	0.035
Death	10 612 (21.7%)	2172 (17.2%)	5367 (21.6%)	3073 (26.8%)	<0.001

Data are *n* (%) or mean (± SD).

^a^Calculated as AI-ECG age minus chronological age.

### Correlation between AI-ECG age and chronological age

**Figure 2:**
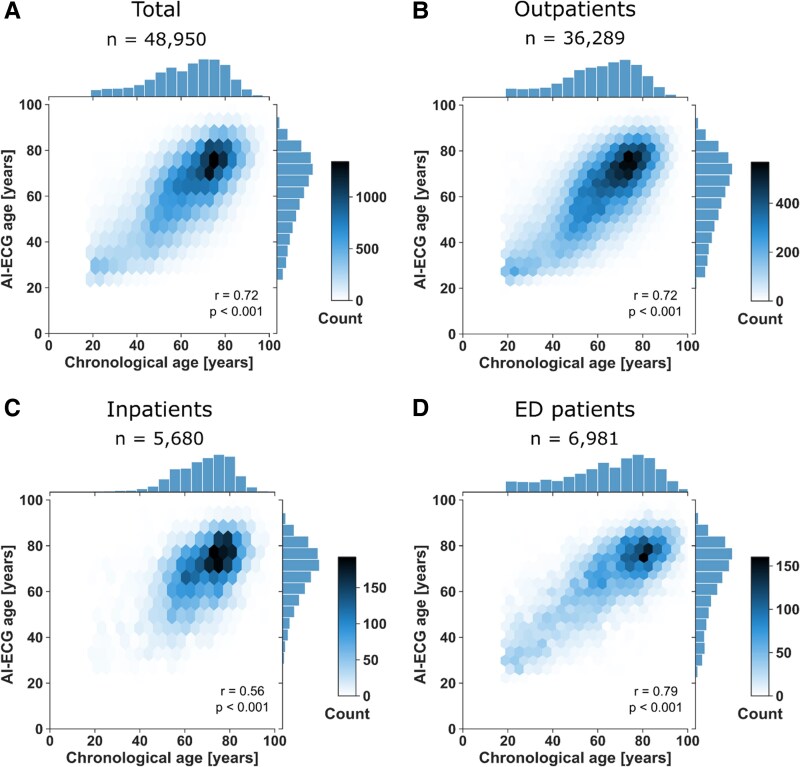
Correlation between AI-ECG age and chronological age across cohorts. Each correlation is visualized using a hexbin plot, where a hexagon represents the density of patients within a region, with darker shading indicating a higher density of patients. The marginal histograms display the distribution of AI-ECG age (*y*-axis) and chronological age (*x*-axis). The subplots correspond to different patient populations: (*A*) The total cohort. (*B*) Outpatients. (*C*) Inpatients. (*D*) Emergency department (ED) patients.

**Figure 3:**
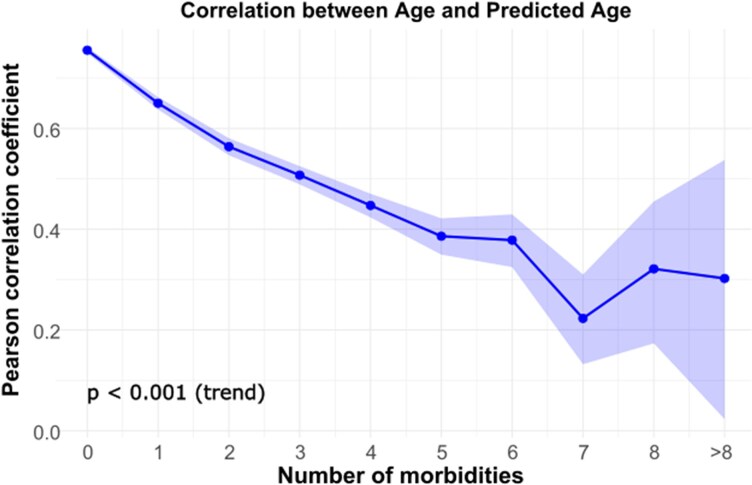
Correlation between AI-ECG age and chronological age across morbidity burden. The curve shows the Pearson correlation coefficient between AI-ECG age and chronological age, stratified by the number of morbidities with 95% CIs displayed. A significant decreasing trend is observed, as confirmed by Fisher’s Z-test, indicating that the correlation weakens as the number of comorbidities increases.

*Figure 2* illustrates the distribution of chronological age and AI-ECG age. Overall, AI-ECG age was strongly correlated with chronological age (Pearson correlation *r* = 0.72, *P* < 0.001). However, substantial variation was observed between the three cohorts (*r* = 0.56 in the inpatient cohort, *r* = 0.79 in the outpatient cohort, and *r* = 0.78 in the ED cohort; Pearson correlation test, *P* < 0.001 for all). The differences between these correlation coefficients were statistically significant (*P* < 0.001, fisher Z-test for comparison of correlation coefficients), likely due to the markedly different prevalence of morbidities across the cohorts. As illustrated in *Figure 3* and detailed in Supplementary material online, *Table S8* the correlation between AI-ECG age and chronological age weakened with an increasing number of morbidities to *r* = 0.30 in those with >8 morbidities. This difference was statistically significant in most pairwise comparisons (*P* < 0.001, Fisher’s Z-test), suggesting that AI-ECG age is increasingly influenced by disease burden rather than solely reflecting chronological ageing. However, the difference between some adjacent morbidity groups (e.g. 8 vs. >8 morbidities, *P* = 0.90) was not statistically significant. A regression analysis demonstrated that both chronological age and the number of comorbidities significantly contributed to the prediction of AI-ECG age (*P* < 0.001). Each additional comorbidity increased the predicted age by an average of 0.97 years, independent of chronological age.

### Association of Δ-age with mortality

**Figure 4:**
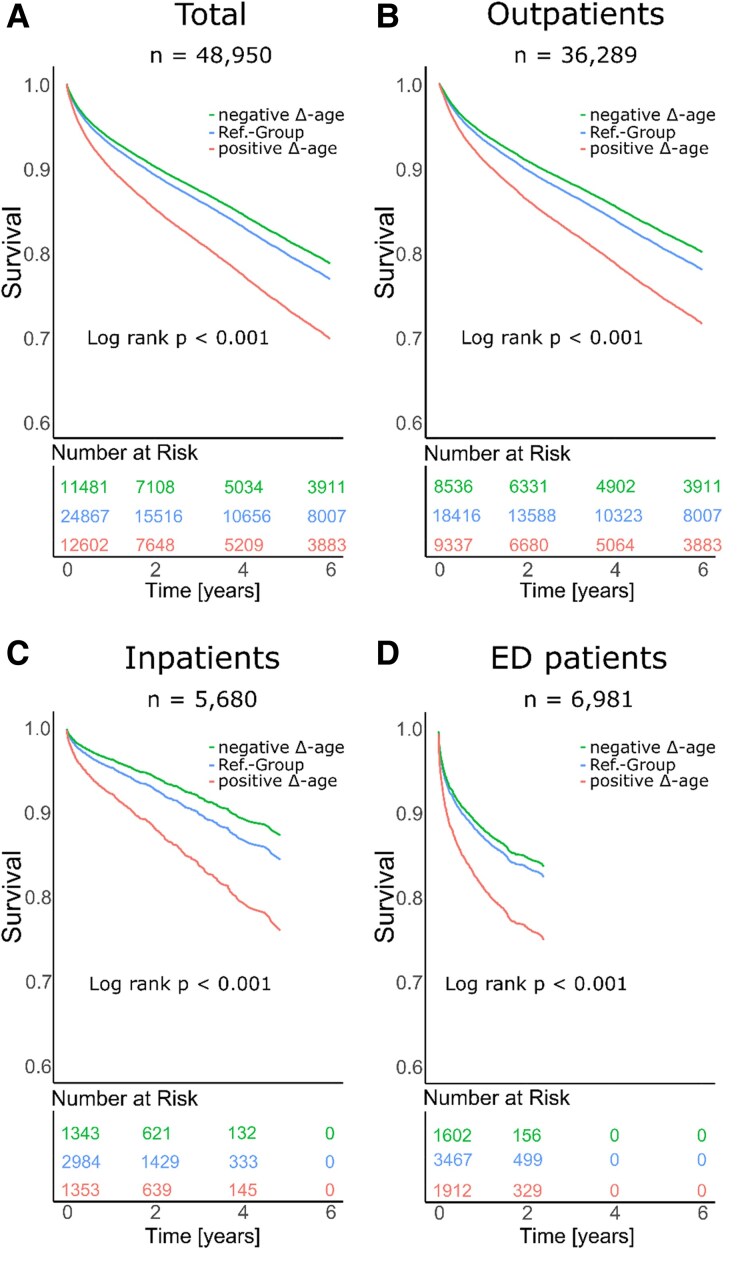
Age- and gender-adjusted Kaplan–Meier survival curves across cohorts. Each Kaplan–Meier survival curve is stratified by AI-ECG Δ-age (negative (≥8 years younger), reference, and positive (≥8 years older). Survival probabilities are adjusted for age and gender. Survival curves are shown for (*A*) the total cohort, (*B*) outpatients, (*C*) inpatients, and (*D*) patients admitted to the medical emergency department (ED). The number of patients at risk is shown below each plot at different time points. Log-rank tests indicate significant differences in survival across Δ-age groups in all cohorts.

In the total cohort, individuals with negative Δ-age had a significantly reduced age-adjusted mortality risk (HR: 0.88, CI: 0.84–0.92, *P* < 0.001), whereas individuals with positive Δ-age had a significantly increased age-adjusted mortality risk (HR: 1.45, CI: 1.37–1.52, *P* < 0.001), both compared with the reference group. *Figure 4* shows the adjusted survival curves for patients stratified according to Δ-age; *Table 2* shows the corresponding hazard ratios.

**Table 2:** Prediction of mortality by categorical Δ-age in different Cox models

Model	Group	Total cohort *n* = 48 950	Outpatients *n* = 36 289	Inpatients *n* = 5680	ED Patients *n* = 6981
		Hazard Ratio (95% CI)	Hazard Ratio (95% CI)	Hazard Ratio (95% CI)	Hazard Ratio (95% CI)
Model 1	Positive Δ-age	1.45 (1.37–1.52) *P* < 0.001	1.41 (1.34–1.49) *P* < 0.001	1.73 (1.32–2.25) *P* = 0.029	1.58 (1.29–1.94) *P* < 0.001
	Negative Δ-age	0.88 (0.84–0.92) *P* < 0.001	0.87 (0.83–0.91) *P* < 0.001	0.78 (0.63–0.98) *P* < 0.001	0.91 (0.79–1.06) *P* = 0.223
Model 2	Positive Δ-age	1.40 (1.32–1.47) *P* < 0.001	1.37 (1.30–1.45) *P* < 0.001	1.62 (1.24–2.12) *P* < 0.001	1.53 (1.25–1.87) *P* < 0.001
	Negative Δ-age	0.90 (0.86–0.94) *P* < 0.001	0.88 (0.84–0.93) *P* < 0.001	0.83 (0.66–1.02) *P* = 0.079	0.93 (0.81–1.08) *P* = 0.348
Model 3	Positive Δ-age	1.28 (1.21–1.35) *P* < 0.001	1.28 (1.20–1.35) *P* < 0.001	1.55 (1.13–2.12) *P* = 0.006	1.32 (1.05–1.66) *P* = 0.016
	Negative Δ-age	0.96 (0.92–1.01) *P* = 0.100	0.97 (0.92–1.02) *P* = 0.227	0.79 (0.60–1.03) *P* = 0.079	0.83 (0.69–0.99) *P* = 0.038
Model 4	Positive Δ-age	1.26 (1.20–1.34) *P* < 0.001	1.26 (1.19–1.34) *P* < 0.001	1.49 (1.09–2.05) *P* = 0.013	1.29 (1.03–1.62) *P* = 0.032
	Negative Δ-age	0.96 (0.92–1.01) *P* = 0.092	0.97 (0.92–1.02) *P* = 0.212	0.81 (0.62–1.07) *P* = 0.132	0.85 (0.71–1.01) *P* = 0.068

ED, emergency department; Cox model 1 adjusted for age and gender; Cox model 2 adjusted for age, gender, arterial hypertension, stroke, coronary artery disease, chronic kidney disease, diabetes mellitus, and atrial fibrillation; Cox model 3 adjusted for age, gender, PQ interval, P duration, QRS duration, QT interval, and RR interval; Cox model 4 is adjusted for all parameters in model 2 and model 3.

After adjustment for cardiovascular risk factors (Cox model 2), the association between Δ-age and mortality risk remained significant in the entire cohort (*Table 2*). A positive Δ-age was associated with an increased risk of death (HR: 1.40, CI: 1.32–1.47, *P* < 0.001), whereas a negative Δ-age was associated with a decreased risk of death (HR: 0.90, CI: 0.86–0.94, *P* < 0.001).

Implementing standard ECG parameters in the Cox models, with or without CV factors (Cox models 3 and 4, respectively) decreased the predictive value of AI-ECG. While positive Δ-age remained significantly associated with increased mortality risk (model 3: HR: 1.28, CI: 1.21–1.35, *P* < 0.001), negative Δ-age was no longer significant (model 3: HR: 0.96, CI: 0.92–1.01, *P* = 0.100).

Similar findings were observed in the subcohorts (*Table 2*, *Figure 4*, and Supplementary material online, *Figure S1*), as well as when Δ-age was used as continuous variable (see Supplementary material online, *Table S9*).

The prognostic value of positive Δ-age remained significant across different age strata, particularly pronounced in younger individuals (under 40 years; Supplementary material online, *Table S10*).

**Figure 5:**
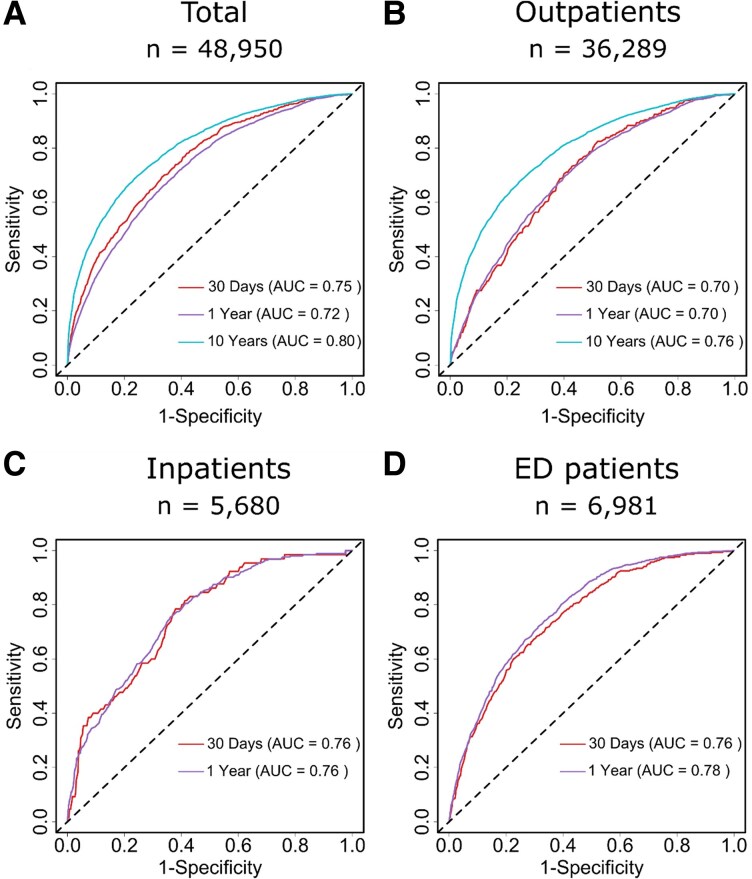
Receiver operating characteristic (ROC) curves for mortality prediction using Δ-age, chronological age, and gender. The ROC curves are shown for (*A*) the total cohort, (*B*) outpatients, (*C*) inpatients, and (*D*) patients admitted to the medical emergency department (ED). The models were evaluated at 30 day, 1 year, and 10 year follow-ups, with the AUC values provided for each time point. The dashed diagonal line represents the line of no discrimination (AUC = 0.5).

Subsequent analyses demonstrated that the different models had robust performance in predicting mortality at different time points. For the entire cohort, model 1 achieved an AUC of 0.75 (CI: 0.74–0.77, *P* < 0.001, DeLong test) at 30 days, 0.72 (CI: 0.72–0.73, *P* < 0.001, DeLong test) at 1 year, and an AUC of 0.80 (CI: 0.80–0–81, *P* < 0.001, DeLong test) at 10 years (*Figure 5*).

In the total cohort, the inclusion of Δ-age improved risk classification with NRI values of 14.6% at 6 years and 19.4% at 10 years (both *P* < 0.001). IDI values, although statistically significant at most time points, remained modest, indicating only slight refinement of the risk distribution. Comprehensive data on IDI and NRI values for all models are presented in *Table 3* and Supplementary material online, *Table S11*, Supplementary material online, *Figure S2*.

**Table 3:** IDI and continuous NRI when adding Δ-age in different Cox models for the total cohort

	Time	IDI	NRI
**Model 1**	30 days	0% (0–0) *P* = 0.594	6.5% (0.4–17.5) *P* = 0.008
	365 days	0.1% (0.1–0.2) *P* = 0.010	8.3% (3.2–14.1) *P* < 0.001
	6 years	0.5% (0.3–0.7) *P* < 0.001	14.6% (8.0–21.2) *P* < 0.001
	10 years	0.7% (0.5–0.9) *P* < 0.001	19.4% (11.0–27.5) *P* < 0.001
**Model 2**	30 days	0% (0.0–0.1) *P* = 0.539	8.8% (1.2–15.9) *P* = 0.013
	365 days	0.3% (0.2–0.4) *P* < 0.001	6.8% (2.8–11.0) *P* < 0.001
	6 years	0.9% (0.7–1.1) *P* < 0.001	11.8% (6.6–17.1) *P* < 0.001
	10 years	1.2% (0.9–1.4) *P* < 0.001	17.2% (9.5–23.3) *P* < 0.001
**Model 3**	30 days	0% (0.0–0.1) *P* = 0.150	3.6% (−5.5–11.6) *P* = 0.225
	365 days	0.3% (0.2–0.4) *P* < 0.001	1.9% (1.4–6.7) *P* = 0.098
	6 years	0.8% (0.6–1.0) *P* < 0.001	7.4% (4.7–12.1) *P* < 0.001
	10 years	1.2% (0.8–1.2) *P* < 0.001	10.8% (7.1–15.5) *P* < 0.001
**Model 4**	30 days	0% (0.0–0.1) *P* = 0.080	4.8% (−5.5–13.4) *P* = 0.163
	365 days	0.3% (0.2–0.4) *P* < 0.001	3.0% (0.0–7.7) *P* = 0.03
	6 years	0.8% (0.6–1.0) *P* < 0.001	8.2% (5.3–11.9) *P* < 0.001
	10 years	1.0% (0.7–1.2) *P* < 0.001	11.4% (8.0–16.3) *P* < 0.001

Cox model 1 incorporated age, gender and Δ-age; Cox model 2 incorporated age, gender, Δ-age, arterial hypertension, stroke, coronary artery disease, chronic kidney disease, diabetes mellitus, and atrial fibrillation; Cox model 3 incorporated age, gender, Δ-age, PQ interval, P duration, QRS duration, QT interval, and RR interval; Cox model 4 incorporated all Parameters from model 2 and model 3.

IDI, integrated discrimination improvement; NRI, net reclassification index.

Spline analysis revealed a J-shaped association between Δ-age and all-cause mortality (see Supplementary material online, *Figure S3*, Supplementary material online, *Table S12*).

### Association of Δ-age with future CV diagnoses

We also assessed the predictive value of Δ-age for future CV diagnoses. Negative Δ-age was associated with lower odds of developing atrial fibrillation (OR: 0.73, CI: 0.57–0.93, *P* = 0.012), coronary artery disease (OR: 0.78, CI: 0.66–0.92, *P* = 0.004), and arterial hypertension (OR: 0.71, CI: 0.60–0.83, *P* < 0.001). In contrast, a positive Δ-age was associated with higher odds of acute coronary syndrome (OR: 1.96, CI: 1.22–3.15, *P* = 0.006) and cardiomyopathy (OR: 1.34, CI: 1.03–1.75, *P* = 0.032). Predictive performance remained moderate, with AUC values ranging between 0.65 and 0.72 (see Supplementary material online, *Table S13*).

### Mechanistic interpretation of AI-ECG age estimates

**Figure 6:**
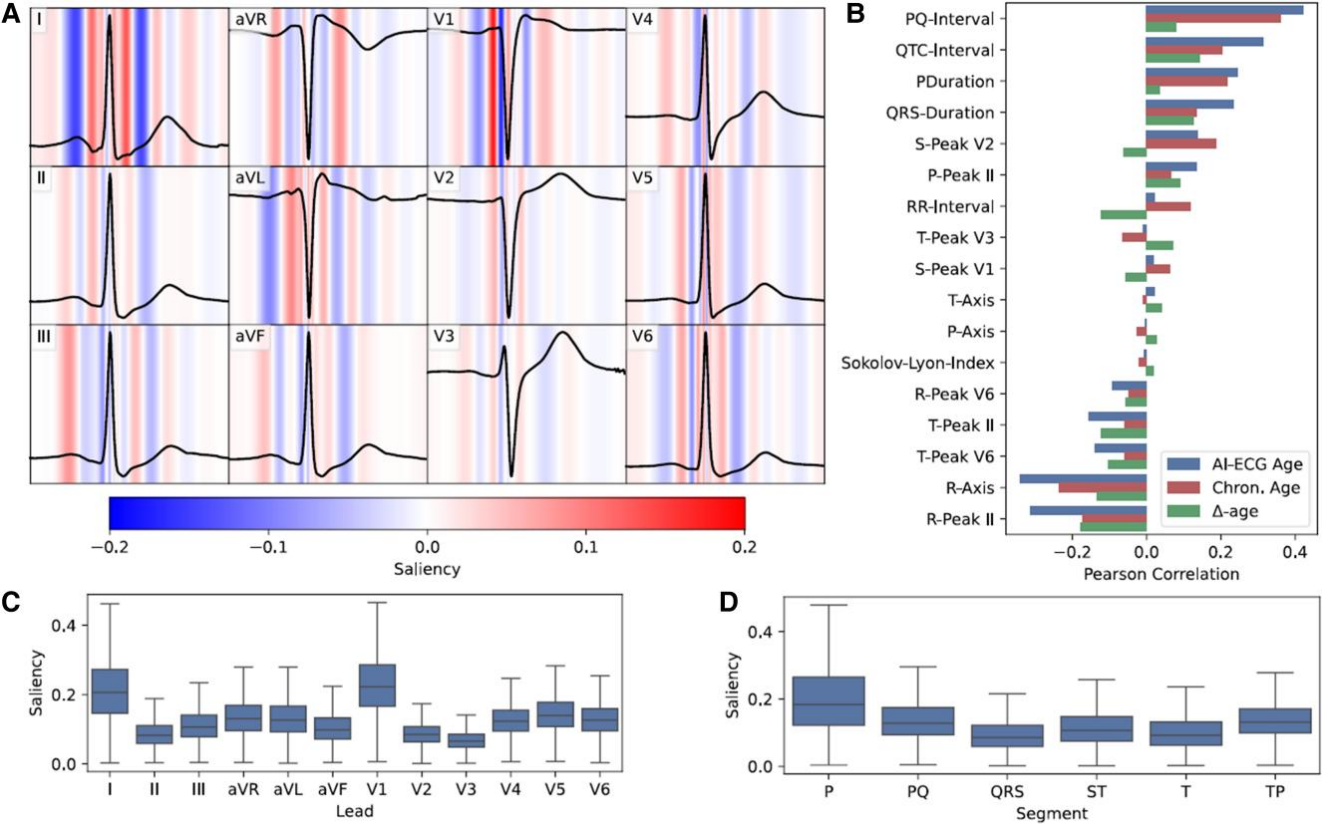
Saliency analysis and correlations of ECG parameters with AI-predicted ECG age, chronological age, and Δ-age. (*A*) Mean saliency across the 12 standard leads for a random subset of 20 000 ECGs. A representative ECG wave is included for visualization purposes. Positive saliency values indicate that an increase in the voltage would increase predicted age, while negative values indicate that a decrease in voltage would increase predicted age. (*B*) Pearson correlation coefficients of selected ECG parameters with AI-predicted ECG age, chronological age, and Δ-age in the complete patient cohort. (*C*) Boxplots of mean absolute saliency by ECG lead, calculated from the same random subset of 20 000 ECGs as panel A. (*D*) Boxplots of mean absolute saliency for individual ECG segments (*P*-wave, PQ interval, QRS complex, ST segment, *T*-wave, and TP interval), also derived from the same subset as panel A.

Exploratory AI analyses are depicted in *Figure 6*. As shown in panel A, the saliency maps demonstrate a wide range of sensitivity across segments and leads. Panels C and D show mean absolute saliency for each lead and segment, respectively. The model appears to be most sensitive to leads I and V1 (Panel C) as well as the *P*-wave, TP and PQ segments (Panel D), although other segments of the ECG are also of importance. Panel B shows the Pearson correlations between AI-ECG age and standard ECG measures. The strongest positive correlations are observed for the PQ interval, QTc interval, and *P* duration, while the strongest negative correlations are seen for the R peak and R axis, as well as the T peak and T axis. Supplementary material online, *Table S14* shows the distribution of standard ECG parameters across Δ-age groups. Detailed data for the subcohorts are available in the supplementary material (see Supplementary material online, *Table S15*, Supplementary material online, *Figures S4* and *S5*).

### Stability of Δ-age

In patients with multiple ECGs, the standard deviation of Δ-age, reflecting its intra-individual variability, was on average 6.45 years (95% CI: 6.39–6.51; Supplementary material online, *Table S16*). The time span between the earliest and latest ECG per patient was on average 5.64 years (2060 days, 95% CI: 2038.61–2082.29), representing the individual observation period. In linear regression, a higher number of hospital admissions during this interval was significantly associated with greater Δ-age variability (β = 0.35, 95% CI: 0.32–0.37, *P* < 0.001).

### Exploratory analysis of the optimal cutoff value for Δ-age

In an exploratory analysis using incremental Δ-age thresholds, 7.5 years was identified as the optimal cutoff for prognostic discrimination (see Supplementary material online, *Figure S6*), closely aligning with the predefined 8 year threshold.

## Discussion

The findings of our study can be summarized as follows: in a large cohort of patients with cardiovascular and acute medical conditions, Δ-age emerged as a strong and independent predictor of all-cause mortality, with consistent prognostic performance across inpatients, outpatients, and ED presentations. Δ-age remained predictive after adjustment for comorbidities and standard ECG features, highlighting its added value beyond traditional risk factors. Importantly, the strength of correlation between AI-ECG age and chronological age declined with increasing comorbidity burden, suggesting that Δ-age may capture not only biological ageing but also overt and potentially subclinical disease processes. We further showed that Δ-age was associated with future cardiovascular diagnoses and that intra-individual fluctuations over time correlate with clinical instability, such as hospital readmissions. Finally, explainable AI methods revealed the *P*-wave as the most influential ECG component for age prediction, reinforcing the biological plausibility of the model and pointing towards atrial conduction as a sensitive marker of cardiovascular ageing.

Compared with previous studies, our cohort represents a population with markedly higher cardiovascular risk. Prior investigations of AI-ECG age have primarily focused on community-based cohorts with low disease burden and low event rates and hospital cohorts with lower-risk patients^11,19,35–39^ (see Supplementary material online, *Table S17*). For instance, Attia *et al*. and Lima *et al*. analysed general population samples with limited cardiovascular comorbidities and reported strong associations between Δ-age and mortality, albeit in predominantly healthy individuals.^22,40^ Cho *et al*. evaluated a hospital population but did not perform a mortality analysis and included mainly low-risk patients.^41^ Hirota *et al*. studied patients with CVD but focused on incident cardiovascular events rather than all-cause mortality.^42^ In contrast, our cohort of 48 950 patients had a high prevalence of established CVD and an all-cause mortality rate exceeding 6%/year during follow-up. This high-risk clinical context allowed for a more stringent test of AI-ECG age as a prognostic tool. Importantly, we showed that Δ-age remained independently predictive of mortality even after adjusting for conventional risk factors and comorbidities, and this held true across all care settings and age strata. These findings substantially expand the existing evidence base and demonstrate the robustness and potential clinical utility of AI-ECG-derived age estimation in real-world, high-risk populations.

Beyond mortality prediction, AI-ECG age may hold significant potential for predicting future CV diagnoses or detecting latent conditions already present at the time of ECG recording.^42^ Previous research by Cho *et al*. has demonstrated that Δ-age can predict the future onset of atrial fibrillation, a finding we were able to confirm in our study.^41^ In our study, Δ-age was also associated with future CV diagnoses, including arterial hypertension, acute, and chronic coronary syndromes and cardiomyopathies. However, the effect sizes were smaller in our acutely ill cohort compared with those reported by Chang *et al*.^20^ This discrepancy may be partly due to methodological limitations, as patients with relevant conditions may not have been followed up at our institution after their initial ECG recording. An important consideration in this context is whether patients were truly disease-free at the time of ECG acquisition or whether underlying conditions were already present but undiagnosed. Addressing this question will require further investigation.

As with most neural networks, the exact mechanisms by which AI models infer biological age from surface ECGs remain largely unknown. To gain insight into this process, we used two different forms of model explainability: the correlation between standard ECG parameters and predicted age and saliency maps. Saliency maps show which regions of the signal the model is most sensitive to, but not why the model is sensitive to them.^43^ Hence, the analysis of more standard ECG measurements provides a valuable counterpart by providing more detail as to why the model might be sensitive to specific segments of the ECG. Both analyses suggest that the model’s predictions are most influenced by the *P*-wave, as well as the PQ and TP segments, regions associated with atrial conduction.^44^ Notably, the limited saliency of high-amplitude components such as the QRS complex suggest that subtle, low-amplitude signals may carry critical age-related information that is otherwise masked by dominant ventricular activity. Prior work by Attia *et al*., identified the *P*-wave and PQ interval as key features in predicting incident atrial fibrillation, an age-related arrhythmia, from sinus rhythm ECGs.^15^ Our results extend these insights by demonstrating significant associations of AI-ECG age with both time-dependent (e.g. P duration, PQ interval) and voltage features (e.g. P peak). While Ott *et al*. showed that *P*-wave morphology influences AI-ECG age in healthy individuals, we confirm and expand this observation in a large, high-risk clinical cohort.^45^ Further support for the role of atrial conduction comes from studies linking *P*-wave indices, such as abnormal axis and advanced interatrial block, to atrial fibrillation risk.^46–48^ Specific orthogonal *P*-wave morphologies have likewise been associated with atrial fibrillation hospitalization in population-based studies.^49^ While our findings emphasize the high significance of atrial conduction, our data alongside with additional studies have indicated that other ECG components also contribute to AI-derived biological age estimations. Although the QRS, ST, and T segments exhibited lower saliency scores, they were not negligible. Their non-zero saliency indicates that these segments still contributed to the prediction, which is further supported by strong correlations with parameters of the R and T segments. Lima *et al*. demonstrated that even ECG tracings classified as normal could reflect subtle, age-related changes across the entire ECG, contributing significantly to mortality predictions.^22^ Similarly further studies identified significant associations between increased biological ECG age and altered ventricular conduction features, including prolonged QRS duration, QT interval, and corrected QT interval (QTc), underscoring the comprehensive nature of ECG-derived age information.^19^ Moreover, advanced ECG parameters, such as QRS complexity and spatial QRS-T angle, had been identified to contribute to heart age from short-duration ECG recordings.^45^ Hempel *et al*. further demonstrated that AI-ECG age is a complex phenomenon, not fully explainable by simple ECG measures alone, as different leads exhibit varying areas of sensitivity.^50^ Additionally, Attia *et al*. showed that deep learning models identify not only traditional ECG parameters but also novel ECG features that significantly enhance the accuracy of biological age estimation.^40^ These studies collectively underscore that multiple ECG segments, beyond atrial-specific features, play a pivotal role in determining biological heart age, highlighting the complexity and integrative nature of AI-driven ECG analysis. Collectively, these observations underscore the biological plausibility that age-related atrial remodelling, including fibrosis and structural changes, may shape AI-ECG-derived age estimates through their effects on conduction alongside other parts of the ECG.

The limitations of our study should be acknowledged. First, its retrospective design introduces inherent methodological constraints. Conducted at a single academic tertiary care centre, the generalizability of our findings to broader or non-tertiary populations may be limited. Although the AI algorithm was externally validated using data from Brazilian cohorts, external validation of our risk prediction findings, particularly in other healthcare settings and ethnic groups, remains necessary. Prospective studies in diverse clinical contexts are needed to confirm and refine risk thresholds. A known limitation of the AI-ECG age algorithm is its tendency to overestimate age in younger individuals and underestimate it in older individuals, which may introduce bias in the distribution of risk factors. Nevertheless, Cox models adjusted for chronological age and cardiovascular risk factors confirmed the prognostic value of AI-ECG age, with significant differences in mortality risk. Follow-up duration varied across cohorts due to technical factors, with shorter observation periods in ED and inpatient populations. This may have limited the detection of long-term outcomes such as chronic disease development. Additionally, future cardiovascular diagnoses could only be captured if patients returned to our institution, and diagnoses were extracted from the in-hospital information system, which may be incomplete. This could lead to a systematic underestimation of comorbidities and attenuate observed associations. However, this limitation likely does not apply to acute coronary syndromes, as our hospital is the sole provider for such cases in the catchment area. Another important limitation is the absence of data on medication use, particularly drugs known to influence ECG parameters such as beta-blockers or antiarrhythmics. Therefore, we cannot exclude potential confounding effects of pharmacological interventions. Future studies should explore whether medication-related ECG changes impact AI-derived age estimates. Furthermore, cause-specific mortality could not be assessed, limiting more granular outcome analyses. Although our primary endpoint was all-cause mortality, future investigations incorporating cause-specific data could provide deeper insight into the predictive utility of Δ-age, especially with regard to cardiovascular mortality. The explainability analysis was performed post hoc on a black box deep learning model, which limits the strength of our conclusions. Since the model’s internal reasoning process is not directly accessible, the explanations produced are approximations and may not faithfully represent its true decision process.^51,52^ Future work should explore interpretable-by-design models,^53^ where the relationship between input signal and predictions is inherently understandable. Finally, external validation in independent populations is essential to confirm the generalizability of our findings, particularly in relation to the identified Δ-age thresholds.

In conclusion, our study shows that Δ-age is a significant and independent prognostic marker in patients with established CVD and those seeking acute care that can be obtained rapidly, easily, and at minimal cost. Its implementation in routine clinical practice could enhance traditional risk stratification methods, enabling more precise identification of high-risk patients. Furthermore, AI-ECG age could serve as a valuable tool for longitudinal monitoring of cardiovascular health, potentially guiding personalized therapeutic interventions aimed at modifying cardiovascular risk factors over time. Despite these findings, the mechanistic basis of AI-ECG age estimation remains incompletely understood and warrants further investigation in future studies to advance both clinical and pathophysiological insights.

## Biography


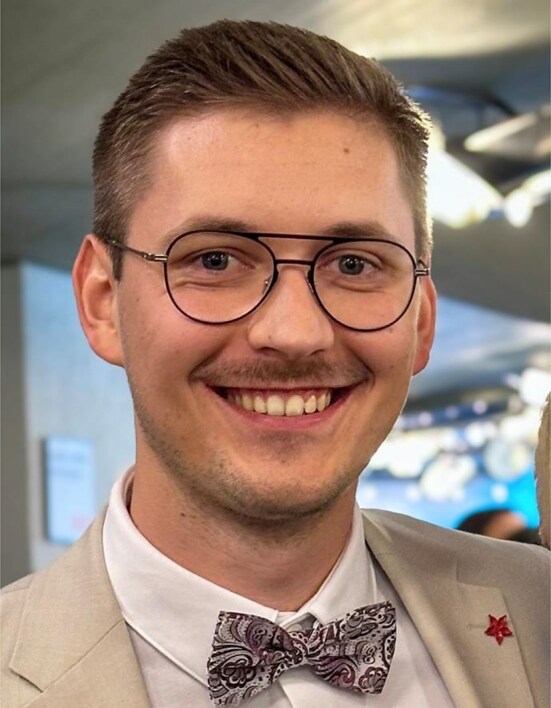


Daniel Pavluk is a first-year PhD student under the supervision of Prof. Axel Bauer at the Department of Cardiology, Medical University of Innsbruck, Austria. He completed his medical degree at the Medical University of Innsbruck in 2024. He is part of a research group focusing on risk stratification in cardiovascular medicine through non-invasive biosignal analysis. His current work investigates the application of artificial intelligence methods to electrocardiographic data, aiming to improve diagnostics and prognostics in clinical and wearable settings.

## Supplementary Material

ztaf109_Supplementary_Data

## Data Availability

The data that support the findings of this study are not publicly available but can be made available upon reasonable request to the corresponding author. The data are located in controlled access data storage at the Medical University of Innsbruck.

## Code availability

The code for the AI-ECG model has been made available by Lima *et al*. (https://github.com/antonior92/ecg-age-prediction).

## References

[1] Roth GA, Mensah GA, Johnson CO, Addolorato G, Ammirati E, Baddour LM, et al Global burden of cardiovascular diseases and risk factors, 1990–2019: update from the GBD 2019 study. J Am Coll Cardiol 2020;76:2982–3021. 33309175 10.1016/j.jacc.2020.11.010 PMC7755038

[2] Arnett DK, Blumenthal RS, Albert MA, Buroker AB, Goldberger ZD, Hahn EJ, et al 2019 ACC/AHA guideline on the primary prevention of cardiovascular disease: a report of the American College of Cardiology/American Heart Association Task Force on Clinical Practice Guidelines. Circulation 2019;140:e596–e646. 30879355 10.1161/CIR.0000000000000678 PMC7734661

[3] Dagenais GR, Leong DP, Rangarajan S, Lanas F, Lopez-Jaramillo P, Gupta R, et al Variations in common diseases, hospital admissions, and deaths in middle-aged adults in 21 countries from five continents (PURE): a prospective cohort study. Lancet 2020;395:785–794. 31492501 10.1016/S0140-6736(19)32007-0

[4] Lanza GA . The electrocardiogram as a prognostic tool for predicting major cardiac events. Prog Cardiovasc Dis 2007;50:87–111. 17765472 10.1016/j.pcad.2007.03.003

[5] Kligfield P, Gettes LS, Bailey JJ, Childers R, Deal BJ, Hancock EW, et al Recommendations for the standardization and interpretation of the electrocardiogram: part I: the electrocardiogram and its technology: a scientific statement from the American Heart Association Electrocardiography and Arrhythmias Committee, Council on Clinical Cardiology; the American College of Cardiology Foundation; and the Heart Rhythm Society: endorsed by the International Society for Computerized Electrocardiology. Circulation 2007;115:1306–1324. 17322457 10.1161/CIRCULATIONAHA.106.180200

[6] Mason JW, Hancock EW, Gettes LS; American Heart Association Electrocardiography and Arrhythmias Committee, Council on Clinical Cardiology; American College of Cardiology Foundation; Heart Rhythm Society, et al Recommendations for the standardization and interpretation of the electrocardiogram: part II: electrocardiography diagnostic statement list: a scientific statement from the American Heart Association Electrocardiography and Arrhythmias Committee, Council on Clinical Cardiology; the American College of Cardiology Foundation; and the Heart Rhythm Society: endorsed by the International Society for Computerized Electrocardiology. Circulation 2007;115:1325–1332. 17322456 10.1161/CIRCULATIONAHA.106.180201

[7] Simonson E . The effect of age on the electrocardiogram. Am J Cardiol 1972;29:64–73. 4257486 10.1016/0002-9149(72)90417-1

[8] Ajmal M, Marcus F. Standardization in performing and interpreting electrocardiograms. Am J Med 2021;134:430–434. 33359812 10.1016/j.amjmed.2020.10.042

[9] Lorenz EC, Zaniletti I, Johnson BK, Petterson TM, Kremers WK, Schinstock CA, et al Physiological age by artificial intelligence-enhanced electrocardiograms as a novel risk factor of mortality in kidney transplant candidates. Transplantation 2023;107:1365–1372. 36780487 10.1097/TP.0000000000004504 PMC10205652

[10] Zworth M, Kareemi H, Boroumand S, Sikora L, Stiell I, Yadav K. Machine learning for the diagnosis of acute coronary syndrome using a 12-lead ECG: a systematic review. CJEM 2023;25:818–827. 37665551 10.1007/s43678-023-00572-5

[11] Muzammil MA, Javid S, Afridi AK, Siddineni R, Shahabi M, Haseeb M, et al Artificial intelligence-enhanced electrocardiography for accurate diagnosis and management of cardiovascular diseases. J Electrocardiol 2024;83:30–40. 38301492 10.1016/j.jelectrocard.2024.01.006

[12] Topol EJ . High-performance medicine: the convergence of human and artificial intelligence. Nat Med 2019;25:44–56. 30617339 10.1038/s41591-018-0300-7

[13] Ribeiro AH, Ribeiro MH, Paixao GMM, Oliveira DM, Gomes PR, Canazart JA, et al Automatic diagnosis of the 12-lead ECG using a deep neural network. Nat Commun 2020;11:1760. 32273514 10.1038/s41467-020-15432-4 PMC7145824

[14] Raghunath S, Ulloa Cerna AE, Jing L, vanMaanen DP, Stough J, Hartzel DN, et al Prediction of mortality from 12-lead electrocardiogram voltage data using a deep neural network. Nat Med 2020;26:886–891. 32393799 10.1038/s41591-020-0870-z

[15] Attia ZI, Noseworthy PA, Lopez-Jimenez F, Asirvatham SJ, Deshmukh AJ, Gersh BJ, et al An artificial intelligence-enabled ECG algorithm for the identification of patients with atrial fibrillation during sinus rhythm: a retrospective analysis of outcome prediction. Lancet 2019;394:861–867. 31378392 10.1016/S0140-6736(19)31721-0

[16] Attia ZI, Kapa S, Lopez-Jimenez F, McKie PM, Ladewig DJ, Satam G, et al Screening for cardiac contractile dysfunction using an artificial intelligence-enabled electrocardiogram. Nat Med 2019;25:70–74. 30617318 10.1038/s41591-018-0240-2

[17] Ladejobi AO, Medina-Inojosa JR, Shelly Cohen M, Attia ZI, Scott CG, LeBrasseur NK, et al The 12-lead electrocardiogram as a biomarker of biological age. Eur Heart J Digit Health 2021;2:379–389. 36713596 10.1093/ehjdh/ztab043 PMC9707884

[18] Lopez-Jimenez F, Kapa S, Friedman PA, LeBrasseur NK, Klavetter E, Mangold KE, et al Assessing biological age: the potential of ECG evaluation using artificial intelligence: JACC family series. JACC Clin Electrophysiol 2024;10:775–789. 38597855 10.1016/j.jacep.2024.02.011

[19] Baek Y-S, Lee D-H, Jo Y, Lee S-C, Choi W, Kim D-H. Artificial intelligence-estimated biological heart age using a 12-lead electrocardiogram predicts mortality and cardiovascular outcomes. Front Cardiovasc Med 2023;10:1137892. 37123475 10.3389/fcvm.2023.1137892 PMC10133724

[20] Chang C-H, Lin C-S, Luo Y-S, Lee Y-T, Lin C. Electrocardiogram-based heart age estimation by a deep learning model provides more information on the incidence of cardiovascular disorders. Front Cardiovasc Med 2022;9:754909. 35211522 10.3389/fcvm.2022.754909 PMC8860826

[21] Rajai N, Medina-Inojosa B, Sheffeh MA, Baez Suarez A, Nyman MA, Attia Z, et al Abstract 13374: effect of moderate to strenuous exercise on biological aging as determined by artificial-enabled electrocardiography. Circulation 2022;146:A13374.

[22] Lima EM, Ribeiro AH, Paixao GMM, Ribeiro MH, Pinto-Filho MM, Gomes PR, et al Deep neural network-estimated electrocardiographic age as a mortality predictor. Nat Commun 2021;12:5117. 34433816 10.1038/s41467-021-25351-7 PMC8387361

[23] World Health Organization . ICD-10: International Statistical Classification of Diseases and Related Health Problems: Tenth Revision. 2nd ed. Geneva: World Health Organization; 2004.

[24] Virtanen P, Gommers R, Oliphant TE, Haberland M, Reddy T, Cournapeau D, et al Scipy 1.0: fundamental algorithms for scientific computing in Python. Nat Methods 2020;17:261–272. 32015543 10.1038/s41592-019-0686-2 PMC7056644

[25] Ribeiro ALP, Paixão GMM, Gomes PR, Ribeiro MH, Ribeiro AH, Canazart JA, et al Tele-electrocardiography and bigdata: the CODE (Clinical Outcomes in Digital Electrocardiography) study. J Electrocardiol 2019;57S:S75–S78. 31526573 10.1016/j.jelectrocard.2019.09.008

[26] He K, Zhang X, Ren S, Sun J . Deep residual learning for image recognition. In: *Proceedings of the 2016 IEEE Conference on Computer Vision and Pattern Recognition (CVPR), Las Vegas, NV, USA, 27 to 30 June 2016*. IEEE.

[27] Simonyan K, Vedaldi A, Zisserman A. Deep inside convolutional networks: visualising image classification models and saliency maps. In: *International Conference on Learning Representations (ICLR), Workshop Track; 2014 April 14 to 16; Banff, Canada*. arXiv: 1312.6034. doi: 10.48550/arXiv.1312.6034.

[28] Makowski D, Pham T, Lau ZJ, Brammer JC, Lespinasse F, Pham H, et al NeuroKit2: a Python toolbox for neurophysiological signal processing. Behav Res Methods 2021;53:1689–1696. 33528817 10.3758/s13428-020-01516-y

[29] R Core Team . *R: A Language and Environment for Statistical Computing*. Vienna, Austria: R Foundation for Statistical Computing; 2024.

[30] Diedenhofen B, Musch J. Cocor: a comprehensive solution for the statistical comparison of correlations. PLoS One 2015;10:e0121945. 25835001 10.1371/journal.pone.0121945 PMC4383486

[31] Denz R, Klaaßen-Mielke R, Timmesfeld N. A comparison of different methods to adjust survival curves for confounders. Stat Med 2023;42:1461–1479. 36748630 10.1002/sim.9681

[32] Wang W, Yan J. Shape-restricted regression splines with R package splines2. J Data Sci 2021;19:498–517.

[33] Blanche P, Dartigues J-F, Jacqmin-Gadda H. Estimating and comparing time-dependent areas under receiver operating characteristic curves for censored event times with competing risks. Stat Med 2013;32:5381–5397. 24027076 10.1002/sim.5958

[34] Inoue E . NRI for risk prediction models with time to event and binary response data. 2018. https://cran.r-project.org/web/packages/nricens/index.html. (Accessed 13 March 2025).

[35] Brant LCC, Ribeiro AH, Pinto-Filho MM, Kornej J, Preis SR, Fetterman JL, et al Association between electrocardiographic age and cardiovascular events in community settings: the Framingham Heart Study. Circ Cardiovasc Qual Outcomes 2023;16:e009821. 37381910 10.1161/CIRCOUTCOMES.122.009821 PMC10524985

[36] Toya T, Ahmad A, Attia Z, Cohen-Shelly M, Ozcan I, Noseworthy PA, et al Vascular aging detected by peripheral endothelial dysfunction is associated with ECG-derived physiological aging. J Am Heart Assoc 2021;10:e018656. 33455414 10.1161/JAHA.120.018656 PMC7955452

[37] Anjewierden S, O’Sullivan D, Mangold KE, Attia IZ, Lopez-Jimenez F, Friedman PA, et al Artificial intelligence-derived electrocardiographic age predicts mortality in adults with congenital heart disease. JACC Adv 2025;4:101777. 40373518 10.1016/j.jacadv.2025.101777 PMC12144430

[38] Wilsgaard T, Rosamond W, Schirmer H, Lindekleiv H, Attia ZI, Lopez-Jimenez F, et al A new biomarker of aging derived from electrocardiograms improves risk prediction of incident cardiovascular disease. JACC Adv 2025;4:101764. 40367762 10.1016/j.jacadv.2025.101764 PMC12142506

[39] Evans S, Howson SA, Booth AEC, Shahmohamadi E, Lim M, Bacchi S, et al Artificial intelligence electrocardiogram-predicted biological age gap and mortality: capturing dynamic risk with multiple electrocardiograms. Heart Rhythm 2025;22:e710–e716. doi: 10.1016/j.hrthm.2025.05.009 9 40368290

[40] Attia ZI, Friedman PA, Noseworthy PA, Lopez-Jimenez F, Ladewig DJ, Satam G, et al Age and sex estimation using artificial intelligence from standard 12-lead ECGs. Circ Arrhythm Electrophysiol 2019;12:e007284. 31450977 10.1161/CIRCEP.119.007284 PMC7661045

[41] Cho S, Eom S, Kim D, Kim TH, Uhm JS, Pak HN, et al Artificial intelligence-derived electrocardiographic aging and risk of atrial fibrillation: a multi-national study. Eur Heart J 2025;46:839–852. 39626169 10.1093/eurheartj/ehae790

[42] Hirota N, Suzuki S, Motogi J, Nakai H, Matsuzawa W, Takayanagi T, et al Cardiovascular events and artificial intelligence-predicted age using 12-lead electrocardiograms. Int J Cardiol Heart Vasc 2023;44:101172. 36654885 10.1016/j.ijcha.2023.101172 PMC9841236

[43] Colin J, Fel T, Cadene R, Serre T. What I cannot predict, I do not understand: a human-centered evaluation framework for explainability methods. Adv Neural Inf Process Syst 2022;35:2832–2845. 37786623 PMC10544769

[44] Platonov PG . Atrial conduction and atrial fibrillation: what can we learn from surface ECG? Cardiol J 2008;15:402–407. 18810714

[45] Ott G, Schaubelt Y, Lopez Alcaraz JM, Haverkamp W, Strodthoff N. Using explainable AI to investigate electrocardiogram changes during healthy aging-from expert features to raw signals. PLoS One 2024;19:e0302024. 38603660 10.1371/journal.pone.0302024 PMC11008906

[46] Filos D, Chouvarda I, Tachmatzidis D, Vassilikos V, Maglaveras N. Beat-to-beat P-wave morphology as a predictor of paroxysmal atrial fibrillation. Comput Methods Programs Biomed 2017;151:111–121. 28946993 10.1016/j.cmpb.2017.08.016

[47] Miles WM . Analysis of P-wave morphology is not passe in this era of high-definition electroanatomic mapping. JACC Clin Electrophysiol 2021;7:1557–1560. 34949423 10.1016/j.jacep.2021.09.018

[48] Baturova MA, Cornefjord G, Carlson J, Johnson LSB, Smith JG, Platonov PG. P-wave characteristics as electrocardiographic markers of atrial abnormality in prediction of incident atrial fibrillation—The Malmo Preventive Project. J Electrocardiol 2024;82:125–130. 38128157 10.1016/j.jelectrocard.2023.12.003

[49] Eranti A, Carlson J, Kentta T, Holmqvist F, Holkeri A, Haukilahti MA, et al Orthogonal P-wave morphology, conventional P-wave indices, and the risk of atrial fibrillation in the general population using data from the Finnish Hospital Discharge Register. Europace 2020;22:1173–1181. 32556298 10.1093/europace/euaa118

[50] Hempel P, Ribeiro AH, Vollmer M, Bender T, Dorr M, Krefting D, et al Explainable AI associates ECG aging effects with increased cardiovascular risk in a longitudinal population study. NPJ Digit Med 2025;8:25. 39806125 10.1038/s41746-024-01428-7 PMC11730300

[51] Arun N, Gaw N, Singh P, Chang K, Aggarwal M, Chen B, et al Assessing the trustworthiness of saliency maps for localizing abnormalities in medical imaging. Radiol Artif Intell 2021;3:e200267. 34870212 10.1148/ryai.2021200267 PMC8637231

[52] Adebayo J, Gilmer J, Muelly M, Goodfellow I, Hardt M, Kim B. Sanity Checks for Saliency Maps. In: Advances in Neural Information Processing Systems 31, NeurIPS 2018, Montréal, Canada. 2018. arXiv:1810.03292.

[53] Rudin C . Stop explaining black box machine learning models for high stakes decisions and use interpretable models instead. Nat Mach Intell 2019;1:206–215. 35603010 10.1038/s42256-019-0048-x PMC9122117

