# Supplementary material for: AI-ECG-derived biological age as a predictor of mortality in cardiovascular and acute care patients
Source: Eur Heart J Digit Health. 2025 Oct 3;6(6):1204–15. doi: 10.1093/ehjdh/ztaf109 (PMC12629650; doi:10.1093/ehjdh/ztaf109)
Supplement: ztaf109_Supplementary_Data [file ztaf109_supplementary_data.pdf]

Supplemental Material

Table S 1: List of all ICD-10 codes that were screened for in the hospital registry, numbers are from the total cohort

ICD-10 code	Absolute frequency (n)	Relative frequency (%)	ICD-10 code	Absolute frequency (n)	Relative frequency (%)
**I10**	19031	38,88%	**I45**	572	1,17%
**I20**	3383	6,91%	**I46**	464	0,95%
**I21**	3957	8,08%	**I47**	1088	2,22%
**I22**	28	0,06%	**I48**	7910	16,16%
**I23**	22	0,04%	**I49**	1704	3,48%
**I24**	184	0,38%	**I50**	2782	5,68%
**I25**	17895	36,56%	**I51**	1484	3,03%
**I26**	1008	2,06%	**I52**	5	0,01%
**I27**	1302	2,66%	**I63**	2827	5,78%
**I28**	10	0,02%	**I64**	208	0,42%
**I30**	107	0,22%	**I70**	3215	6,57%
**I31**	516	1,05%	**I71**	1237	2,53%
**I32**	332	0,68%	**I95**	410	0,84%
**I33**	120	0,25%	**I96**	0	0,00%
**I34**	2919	5,96%	**I97**	53	0,11%
**I35**	3197	6,53%	**I98**	23	0,05%
**I36**	368	0,75%	**I99**	62	0,13%
**I37**	85	0,17%	**N18**	4450	9,09%
**I38**	111	0,23%	**E10**	618	1,26%
**I39**	7	0,01%	**E11**	4999	10,21%
**I40**	124	0,25%	**E13**	104	0,21%
**I41**	1	0,00%	**E14**	1875	3,83%
**I42**	2219	4,53%	**E66**	3204	6,55%
**I43**	16	0,03%	**E78**	11804	24,11%
**I44**	2238	4,57%			

Table S 2: Follow-up durations for different cohorts

Cohort	Median follow-up duration [days]
Total cohort (n = 48,950)	1,167 (CI: 1,144–1,189)
Outpatient cohort (n = 36,289)	2,539 (CI: 2,506–2,588)
Inpatient cohort (n = 5,680)	385 (CI: 368–399)
Emergency department (n = 6,981)	88 (CI: 79–94)

Table S 3: Number of Patients with Available Follow-up Data at 1, 3, 5, and 10 Years by Cohort

Cohort	>1	>3	>5	>10
Total cohort (n = 48,950)	33,877	25,077	20,300	6,617
Outpatient cohort (n = 36,289)	29,811	24,358	20,300	6,617
Inpatient cohort (n = 5,680)	2,923	719	0	0
Emergency department (n = 6,981)	1,143	0	0	0

Table S 4: Baseline characteristics for outpatients n = 36,289

	Total n = 36,289	Positive Δ-age n = 9,337	Reference-Group n = 18,416	Negative Δ-age n = 8,536	*p*-value
Chronological age (years)	60.87 (16.50)	52.71 (15.40)	61.32 (16.50)	68.81 (13.30)	<0.001
AI-ECG age (years)	61.3 (16.30)	68.31 (14.80)	61.46 (16.40)	53.30 (13.6)	<0.001
Δ-age (years)[1]	0.43 (12.30)	15.61 (6.90)	0.13 (4.47)	-15.52 (6.45)	<0.001
Female	16,731 (46.10%)	3,641 (39.00%)	8,485 (46.10%)	4,605 (53.90%)	<0.001
Arterial hypertension	12,972 (35.75%)	3,040 (32.56%)	6,687 (36.31%)	3,245 (38.02%)	<0.001
Diabetes mellitus	3,993 (11.00%)	1,130 (12.10%)	2,054 (11.15%)	809 (9.48%)	<0.001
Dyslipidemia	7,068 (19.48%)	1,668 (17.86%)	3,679 (19.98%)	1,721 (20.16%)	<0.001
Coronary artery disease	11,961 (32.96%)	2,963 (31.73%)	6,126 (33.26%)	2,872 (33.65%)	0.008

Chronic kidney disease	3,509 (9.67%)	980 (10.50%)	1,673 (9.08%)	856 (10.03%)	<0.001
Previous myocardial infarction	2,555 (7.01%)	647 (6.93%)	1,325 (7.19%)	583 (6.83%)	0.482
Heart failure	2,264 (6.24%)	541 (5.79%)	1,134 (6.16%)	589 (6.90%)	0.008
Peripheral arterial disease	2,325 (6.41%)	580 (6.21%)	1,198 (6.51%)	547 (6.41%)	0.636
Previous stroke	1,844 (5.08%)	372 (3.98%)	946 (5.14%)	526 (6.16%)	<0.001
Atrial fibrillation	6,265 (17.26%)	1,603 (17.17%)	3,232 (17.55%)	1,430 (16.75%)	0.303
Death	9,167 (25.3%)	1,943 (20.8%)	4,637 (25.2%)	2,587 (30.3%)	<0.001

Data are n (%) or mean (± SD); [1]calculated: AI estimated ECG age - chronological age

Table S 5: Baseline characteristics for inpatients n = 5,680

	**Total n = 5,680**	**Positive Δ-age n = 1,343**	**Reference-Group n = 2,984**	**Negative Δ-age n = 1,353**	***p*-value**
Chronological age (years)	67.94 (12.50)	59.09 (12.20)	69.44 (11.20)	73.34 (11.00)	<0.001
AI-ECG age (years)	68.02 (12.80)	74.32 (10.8)	69.59 (11.30)	58.06 (12.20)	<0.001
Δ-age (years)[1]	0.94 (11.80)	15.24 (6.51)	0.15 (4.43)	-15.27 (6.27)	<0.001
Female	1,998 (35.14%)	350 (25.80%)	1,067 (35.80%)	581 (43.30%)	<0.001
Arterial hypertension	3,570 (62.85%)	786 (57.84%)	1,921 (64.38%)	863 (64.26%)	<0.001
Diabetes mellitus	1,020 (17.96%)	275 (20.24%)	521 (17.46%)	224 (16.68%)	0.505
Dyslipidemia	3,331 (58.64%)	730 (53.72%)	1,761 (59.01%)	840 (62.55%)	<0.001
Coronary artery disease	4,626 (81.43%)	1,028 (75.64%)	2,466 (82.64%)	1,132 (84.29%)	<0.001

Chronic kidney disease	448 (7.89%)	81 (5.96%)	259 (8.68%)	108 (8.04%)	<0.001
Previous myocardial infarction	861 (15.16%)	212 (15.60%)	455 (15.25%)	194 (14.45%)	0.778
Heart failure	246 (4.33%)	53 (3.90%)	124 (4.16%)	69 (5.14%)	<0.001
Peripheral arterial disease	426 (7.50%)	102 (7.51%)	214 (7.17%)	110 (8.19%)	0.002
Previous stroke	94 (1.65%)	22 (1.62%)	42 (1.41%)	30 (2.23%)	<0.001
Atrial fibrillation	951 (16.71%)	237 (17.44%)	477 (15.99%)	237 (17.65%)	<0.001
Death	466 (8.20%)	81 (5.99%)	258 (8.65%)	127 (9.46%)	<0.001
Data are n (%) or mean (± SD); [1]calculated: AI estimated ECG age - chronological age					

Table S 6: Baseline characteristics for emergency department patients n = 6,981

	**Total n = 6,981**	**Positive Δ-age n = 1,912**	**Reference-Group n = 3,467**	**Negative Δ-age n = 1,602**	***p*-value**
Chronological age (years)	64.35 (19.40)	49.95 (16.90)	65.68 (17.60)	78.66 (12.9)	<0.001
AI-ECG age (years)	65.29 (15.70)	65.48 (15.90)	65.72 (16.50)	64.12 (13.20)	<0.001
Δ-age (years)[1]	0.94 (12.0)	15.53 (6.72)	0.04 (4.55)	-14.55 (5.67)	<0.001
Female	3,489 (49.98%)	794 (41.50%)	1,758 (50.70%)	937 (58.50%)	<0.001
Arterial hypertension	2,489 (35.65%)	484 (25.31%)	1,294 (37.32%)	711 (44.38%)	<0.001
Diabetes mellitus	767 (10.99%)	201 (10.51%)	396 (11.42%)	170 (10.61%)	0.504
Dyslipidemia	1,405 (20.13%)	312 (16.32%)	724 (20.88%)	369 (23.03%)	<0.001
Coronary artery disease	1,309 (18.75%)	308 (16.11%)	647 (18.66%)	354 (22.10%)	<0.001

Chronic kidney disease	493 (7.06%)	62 (3.24%)	256 (7.38%)	175 (10.92%)	<0.001
Previous myocardial infarction	552 (7.92%)	157 (8.21%)	267 (7.70%)	130 (8.11%)	0.777
Heart failure	272 (3.90%)	38 (1.99%)	111 (3.20%)	123 (7.68%)	<0.001
Peripheral arterial disease	464 (6.65%)	100 (5.23%)	233 (6.72%)	131 (8.18%)	0.002
Previous stroke	889 (12.73%)	180 (9.41%)	465 (13.41%)	244 (15.23%)	<0.001
Atrial fibrillation	696 (9.97%)	115 (6.01%)	335 (9.66%)	246 (15.36%)	<0.001
Death	979 (14.0%)	148 (7.74%)	472 (13.6%)	359 (22.4%)	<0.001
Data are n (%) or mean (± SD); [1]calculated: AI estimated ECG age - chronological age					

Table S 7: Discharge diagnoses in ED patients (n = 6,981)

**Specialty**	**Absolute frequency (n)**	**Relative frequency (%)**
Cardiology	2,720	19.23
Endocrinology	1,700	12.02
Allergology/Immunology	1,463	10.34
Rheumatology	1,398	9.88
Gastroenterology	1,266	8.95
Oncology	1,255	8.87
Hematology	1,243	8.86
Psychiatry	1,215	8.59
Nephrology	1,190	8.41
Neurology	1,150	8.13
Pulmonology	981	6.94
Infectiology	537	3.80

Table S 8: Impact of morbidities on Pearson correlation coefficient between AI-ECG age and chronological age

Number of morbidities[1]	Pearson correlation coefficient	*p*-value
0 (n = 19,548)	0.760	< 0.001

1 (n = 8,480)	0.655	< 0.001
2 (n = 6,222)	0.565	< 0.001
3 (n = 6,458)	0.510	< 0.001
4 (n = 4,434)	0.447	< 0.001
5 (n = 2,133)	0.386	< 0.001
6 (n = 1,031)	0.378	< 0.001
7 (n = 438)	0.223	< 0.001
8 (n = 157)	0.321	< 0.001
>8 (n = 49)	0.302	0.035
[1]morbidities defined in the methods section		

Table S 9: Prediction of mortality by Δ-age as continuous variable in different Cox models

Model	Total Cohort n = 48,950	Outpatients n = 36,289	Inpatients n = 5,680	ED Patients n = 6,981
	Hazard Ratio (95% CI) for 1 year increase in Δ-age			
Model 1	1.02 (1.01 – 1.02) p < 0.001	1.02 (1.01-1.02) p < 0.001	1.02 (1.01-1.03) p < 0.001	1.02 (1.01-1.03) p < 0.001
Model 2	1.01 (1.01 – 1.02) p < 0.001	1.01 (1.01 – 1.02) p < 0.001	1.01 (1.01 – 1.03) p = 0.002	1.01 (1.01 – 1.02) p = 0.144
Model 3	1.01 (1.01 – 1.01) p < 0.001	1.01 (1.01 – 1.01) p < 0.001	1.01 (1.01 – 1.01) p < 0.001	1.02 (1.01 – 1.03) p < 0.001
Model 4	1.01 (1.01 – 1.01) p < 0.001	1.01 (1.01 – 1.01) p < 0.001	1.01 (1.00–1.03) p = 0.030	1.02 (1.01 – 1.03) p = 0.071
ED – Emergency department; Cox model 1 adjusted for age and gender; Cox model 2 adjusted for age, gender, arterial hypertension, stroke, coronary artery disease, chronic kidney disease, diabetes mellitus, atrial fibrillation; Cox model 3 adjusted for age, gender, PQ-interval, P-duration, QRS-duration, QT-interval and RR-interval; Cox model 4 is adjusted for all parameters in model 2 and model 3.				

Table S 10: Prediction of mortality by categorical Δ-age in different Cox models and age -groups

Model	Group	Under 40 yrs n = 5,492	40 – 60 yrs n = 14,891	Over 60 yrs n = 28,567
		Hazard Ratio (95% CI)	Hazard Ratio (95% CI)	Hazard Ratio (95% CI)
Model 1	Positive Δ-age	2.11 (1.57 – 2.84) p < 0.001	1.43 (1.29-1.57) p < 0.001	1.44 (1.36-1.54) p < 0.001

	Negative Δ-age	1.57 (0.71 – 3.44)<br>p = 0.263	0.71 (0.60-0.83)<br>p < 0.001	0.88 (0.83-0.92)<br>p < 0.001
Model 2	Positive Δ-age	1.93 (1.43 – 2.61)<br>p < 0.001	1.38 (1.24-1.54)<br>p < 0.001	1.39 (1.31-1.48)<br>p < 0.001
	Negative Δ-age	1.77 (0.81 – 3.90)<br>p = 0.157	0.72 (0.62-0.85)<br>p < 0.001	0.89 (0.85-0.94)<br>p < 0.001
Model 3	Positive Δ-age	1.83 (1.35 – 2.49)<br>p < 0.001	1.38 (1.24–1.54)<br>p < 0.001	1.39 (1.31–1.48)<br>p < 0.001
	Negative Δ-age	1.59 (0.72 – 3.50)<br>p = 0.248	0.72 (0.62–0.85)<br>p < 0.001	0.89 (0.85–0.94)<br>p < 0.001
Model 4	Positive Δ-age	1.77 (1.29 – 2.41)<br>p < 0.001	1.17 (1.04–1.31)<br>p = 0.006	1.29 (1.20–1.37)<br>p < 0.001
	Negative Δ-age	1.75 (0.79 – 3.87)<br>p = 0.164	0.77 (0.65 – 0.90)<br>p = 0.001	0.96 (0.91–1.01)<br>p = 0.114
ED – Emergency department; Cox model 1 adjusted for age and gender; Cox model 2 adjusted for age, gender, arterial hypertension, stroke, coronary artery disease, chronic kidney disease, diabetes mellitus, atrial fibrillation; Cox model 3 adjusted for age, gender, PQ-interval, P-duration, QRS-duration, QT-interval and RR-interval; Cox model 4 is adjusted for all parameters in model 2 and model 3.				

Table S 11: IDI and continuous NRI when adding Δ-age for Cox model 1 across different cohorts

	time	IDI	NRI
total cohort<br>n = 48,950	30 days	0% (0 - 0) p = 0.594	3.1% (-0.1-7.6) p = 0.055
	365 days	0.1% (0.1 - 0.2) p = 0.010	4.8% (2.5-6.5) p < 0.001
	6 years	0.5% (0.3 - 0.7) p < 0.001	9.2% (7.5-12.7) p < 0.001
	10 years	0.7% (0.5 - 0.9) p < 0.001	10.0% (7.5-12.7) p < 0.001
outpatient clinics<br>n = 36,289	30 days	0% (0.0 - 0.0) p = 0.459	7.6% (1.5-12.6) p = 0.010
	365 days	0.1% (0.0 - 0.2) p = 0.005	5.1% (2.5-7.2) p = 0.005
	6 years	0.4% (0.3 - 0.6) p < 0.001	10.0% (7.7-11.4) p < 0.001
	10 years	0.6% (0.4 - 0.8) p < 0.001	10.7% (7.8-12.7) p < 0.001
Inpatient clinics<br>n = 5,680	30 days	0.0% (-0.2 - 0.1) p = 0.389	-3.3% (-14.3-8.5) p = 0.589
	365 days	0.1% (-0.1 - 0.5) p = 0.264	8.6% (1.8-16.7) p = 0.010
ED<br>n = 6,981	30 days	0.0% (-0.1 – 0.3) p = 0.623	3.2% (-5.8-14.3) p = 0.589
	365 days	0.3% (0.0 - 0.5) p = 0.070	3.5% (-3.0-13.5) p = 0.299
IDI – integrated discrimination improvement; NRI – net reclassification index; ED – emergency department			

Cox model 1 incorporates Age and Gender

Table S 12: Results from spline-based Cox regression analysis (adjusted for age and gender)

Variable	Hazard Ratio (95% CI)	p-value
ns(Δ-age, df=3) - Term 1	1.31 (1.01–1.70)	0.042
ns(Δ-age, df=3) - Term 2	1.93 (0.56–6.66)	0.299
ns(Δ-age, df=3) - Term 3	22.84 (11.97–43.59)	<0.001

Table S 13: Odds Ratio and AUC for future CV events in the total cohort

	**Positive Δ-age**		**Negative Δ-age**		**AUC**
	OR	*p*-value	OR	*p*-value	
Atrial fibrillation	1.34 (1.03-1.75)	0.032	0.73 (0.57-0.93)	0.012	0.70
Arterial hypertension	1.17 (0.98-1.39)	0.078	0.71 (0.60-0.83)	< 0.001	0.67
Coronary artery disease	1.10 (0.91-1.32)	0.316	0.78 (0.66-0.92)	0.004	0.68
Acute coronary syndrome	1.96 (1.22-3.15)	0.006	0.78 (0.43-1.43)	0.428	0.68
Cardiomyopathy	1.34 (1.03-1.75)	0.032	0.73 (0.57-0.93)	0.013	0.65
Heart failure	1.48 (0.87-2.51)	0.150	0.81 (0.51-1.30)	0.379	0.72
AUC – area under the curve; CV – cardiovascular; OR – odds ratio					

Table S 14: Standard ECG parameters according to Δ-age in the total population

	**Total n = 48,950**	**Positive Δ-age n = 12,602**	**Reference-Group n = 24,867**	**Negative Δ-age n = 11,481**	***p*-value**
Ventricular Rate [bpm]	73.78 (73.61 - 73.95)	76.68 (76.39-76.96)	72.96 (72.76-73.15)	71.28 (70.98-71.59)	< 0.001
PQ Interval [ms]	167.04 (166.78 - 167.29)	167.19 (166.43-167.96)	168.33 (167.75-168.91)	164.41 (163.58-165.25)	< 0.001
P Duration [ms]	111.07 (110.91 - 111.24)	111.53 (111.16-111.9)	111.4 (111.13-111.67)	110.27 (109.89-110.66)	< 0.001
QRS Duration [ms]	99.08 (98.89 - 99.27)	99.1 (98.73-99.47)	99.61 (99.33-99.89)	98.4 (97.98-98.81)	< 0.001

QT Interval [ms]	437.22 (436.85 - 437.58)	439.86 (439.26-440.46)	437.16 (436.72-437.6)	434.28 (433.62-434.95)	< 0.001
QTc Interval [ms]	400.41 (399.93 - 400.89)	394.52 (393.82-395.23)	402.05 (401.53-402.57)	405.04 (404.22-405.85)	< 0.001
PP Interval [ms]	838.73 (834.21 - 843.25)	801.73 (798.37-805.1)	840.44 (837.92-842.96)	864.28 (856.05-872.51)	< 0.001
P Axis [deg]	57.13 (56.86 - 57.41)	58.54 (58.03-59.05)	57.17 (56.81-57.53)	56.1 (55.58-56.61)	< 0.001
R Axis [deg]	39.67 (39.15 - 40.19)	37.08 (36.16-38.0)	38.08 (37.43-38.72)	46.66 (45.76-47.55)	< 0.001
T axis [deg]	52.18 (51.62 - 52.75)	55.24 (54.36-56.12)	52.48 (51.88-53.09)	48.49 (47.61-49.36)	< 0.001

Data are mean (95% CI).

Table S 15: Comparison of ECG Parameter across groups and cohorts

	**Outpatient clinics n = 36,289**					**Inpatients Clinics n = 5,680**					**ED n = 6,981**				
	**Total n = 36,289**	**Positive Δ-age n = 9,337**	**Reference -Group n = 18,416**	**Negative Δ-age n = 8,536**	***p*-value**	**Total n = 5,680**	**Positive Δ-age n = 1,343**	**Reference -Group n = 2,984**	**Negative Δ-age n = 1,353**	***p*-value**	**Total n = 6,981**	**Positive Δ-age n = 1,912**	**Reference -Group n = 3,467**	**Negative Δ-age n = 1,602**	***p*-value**
**Ventricular rate [bpm]**	73.01 (72.74-73.28)	76.05 (75.74-76.36)	72.55 (72.34-72.77)	70.69 (70.36-71.02)	< 0.001	69.9 (69.22-70.58)	73.6 (72.81-74.4)	69.42 (68.91-69.92)	67.28 (66.42-68.14)	< 0.001	79.1 (78.28-79.91)	81.91 (81.02-82.81)	78.15 (77.52-78.78)	77.79 (76.74-78.83)	< 0.001
**PQ interval [ms]**	166.83 (165.98-167.68)	167.59 (166.64-168.53)	167.9 (167.19-168.61)	163.69 (162.67-164.7)	< 0.001	173.47 (171.88-175.05)	174.27 (172.41-176.14)	175.53 (174.17-176.88)	168.13 (166.38-169.87)	< 0.001	163.74 (162.3-165.18)	160.53 (159.14-161.93)	164.66 (163.51-165.8)	165.58 (163.62-167.53)	< 0.001
**P duration [ms]**	110.15 (109.76-110.54)	110.54 (110.09-110.99)	110.32 (110.0-110.64)	109.34 (108.89-109.8)	< 0.001	115.64 (114.78-116.49)	117.27 (116.25-118.29)	115.94 (115.24-116.64)	113.34 (112.34-114.33)	< 0.001	113.24 (112.51-113.97)	112.58 (111.82-113.34)	113.58 (112.98-114.18)	113.29 (112.35-114.22)	0.03
**QRS duration [ms]**	98.83 (98.42-99.23)	99.11 (98.66-99.56)	99.2 (98.87-99.54)	97.71 (97.23-98.19)	< 0.001	101.74 (100.69-102.79)	101.41 (100.3-102.53)	102.4 (101.54-103.27)	100.61 (99.3-101.92)	< 0.001	99.02 (98.25-99.79)	97.42 (96.74-98.11)	99.36 (98.75-99.98)	100.18 (99.08-101.27)	0.27
**QT interval [ms]**	434.22 (433.61-434.83)	437.69 (437.01-438.38)	434.02 (433.52-434.53)	430.86 (430.14-431.59)	< 0.001	443.77 (442.06-445.48)	445.22 (443.24-447.2)	444.59 (443.25-445.94)	440.52 (438.43-442.61)	< 0.001	447.17 (445.72-448.61)	446.63 (445.11-448.14)	447.41 (446.3-448.52)	447.28 (445.35-449.2)	0.18
**QTc interval [ms]**	398.93 (398.20-399.66)	393.7 (392.9-394.5)	399.84 (399.25-400.43)	402.7 (401.8-403.61)	< 0.001	417.13 (415.2-419.05)	407.36 (405.18-409.54)	418.85 (417.33-420.36)	423.02 (420.62-425.43)	< 0.001	397.33 (395.47-399.18)	389.44 (387.55-391.34)	399.33 (397.9-400.77)	402.4 (399.89-404.91)	< 0.001
**PP interval [ms]**	842.12 (838.59-845.64)	806.97 (803.17-810.77)	847.07 (844.23-849.9)	869.87 (865.44-874.3)	< 0.001	843.78 (828.5-859.06)	791.77 (770.32-813.22)	853.0 (841.88-864.12)	875.07 (859.25-890.89)	< 0.001	774.23 (762.94-785.52)	761.35 (751.0-771.69)	774.7 (764.87-784.52)	788.6 (773.8-803.39)	< 0.001

**P axis [deg]**	57.74 (57.21-58.27)	59.14 (58.51-59.76)	57.57 (57.15-58.0)	56.56 (55.95-57.17)	< 0.001	57.36 (55.73-58.99)	57.02 (54.29-59.74)	57.74 (56.55-58.92)	56.87 (56.05-57.7)	0.099	55.22 (53.97-56.48)	55.89 (54.57-57.22)	55.04 (53.64-56.43)	54.83 (54.05-55.6)	0.0622
**R axis [deg]**	42.76 (41.84-43.68)	38.86 (37.77-39.96)	41.19 (40.43-41.95)	50.42 (49.4-51.43)	< 0.001	35.71 (32.02-39.4)	39.77 (33.86-45.67)	34.32 (31.3-37.34)	34.74 (33.16-36.32)	< 0.001	34.87 (32.74-36.99)	49.45 (47.27-51.63)	32.88 (30.51-35.24)	21.76 (20.37-23.15)	< 0.001
**T axis [deg]**	51.31 (50.45-52.17)	54.84 (53.82-55.87)	51.63 (50.94-52.33)	46.76 (45.8-47.72)	< 0.001	59.87 (56.4-63.34)	64.2 (58.64-69.76)	59.65 (56.94-62.36)	56.06 (54.16-57.97)	< 0.001	52.21 (50.57-53.84)	50.11 (48.59-51.62)	52.55 (50.79-54.31)	53.97 (52.5-55.45)	0.1105
ED – emergency department; Data are mean (95% CI)															

Table S 16: Temporal stability of Δ-age in Patients where time between ECGs is at least 1 year

Variable	Mean (95% CI)
Number of ECGs	6.43 (6.32–6.54)
Time range (days) between ecgs	2060.45 (2038.61–2082.29)
SD of Δ-age	6.45 (6.39–6.51)
Range of Δ-age	15.55 (15.39–15.70)
Mean Δ-age	1.56 (1.38–1.73)
Hospital admissions between ECGs	1.78 (1.74 – 1.81)

Table S 17: Overview on studies with AI-ECG age

Author	Reference	N	Pat. Char.	Mortality/year
Toya et al.	(36)	531	Hospital cohort	3.9%
Lima et al.	(22)	1,558,415	General population	0.1%
Lindow et al.	(54)	2,771	General population and cardiovascular Patients	N/A
Libiseller-Egger et al.	(55)	34,432	General population	N/A
Hirota et al.	(42)	17,042	Hospital cohort, cardiovascular primary care Center	3.0%
Baek et al.	(19)	34,317	Health examination center	0.75%
Park et al.	(56)	5,729	Hospital cohort, cardiovascular patients	N/A
Cho et al.	(57)	280,612	General population, Hospital cohort	N/A
Wilsgaard et al.	(38)	7,108	General population	0.49%
Anjewierden et al.	(37)	5,780	Hospital cohort, adults with congential heart disease	2.26%
Evans et al.	(39)	46,960	Hospital cohort, cardiovascular patients	2.76%

**Pavluk et.al.**		**48,950**	**Hospital cohort, cardiovascular patients and ED Patients**	**6.58%**

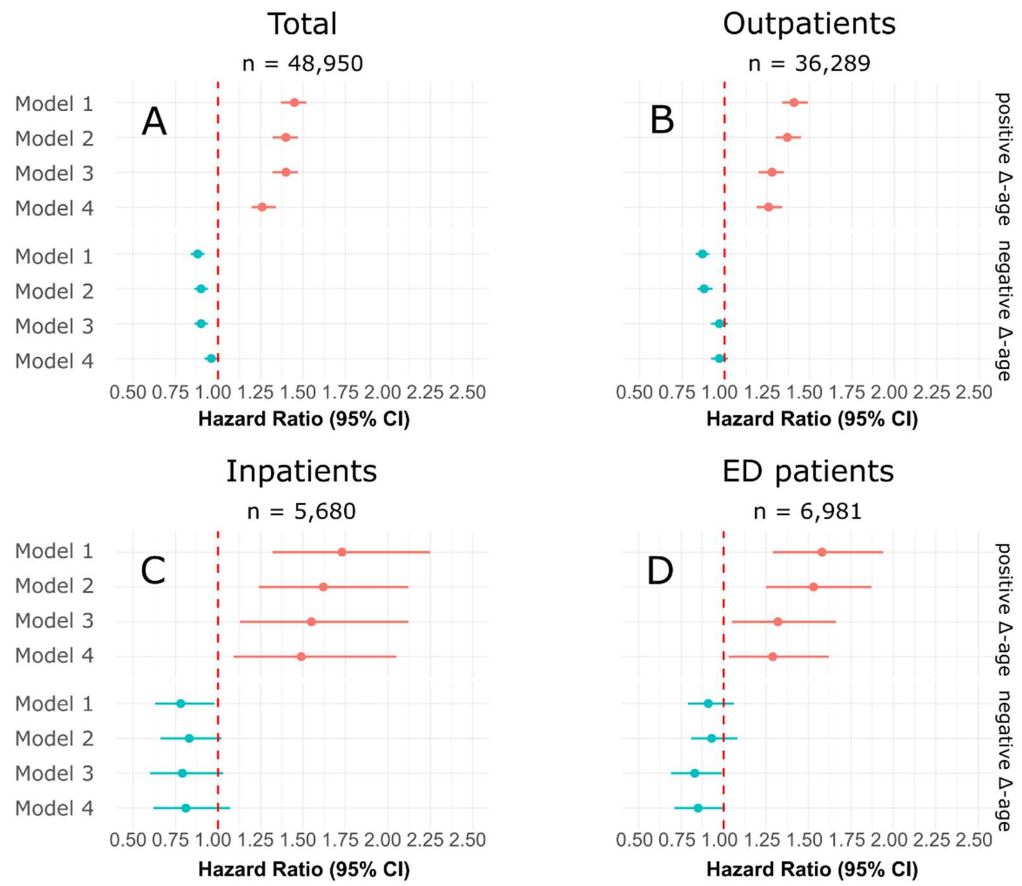

Figure S 1: Forest plot displaying Hazard Ratios (HR) with 95% CI for Cox models with different adjustments. Panel A shows total cohort, panel B outpatients, panel C inpatients and panel D patients admitted to the medical emergency department

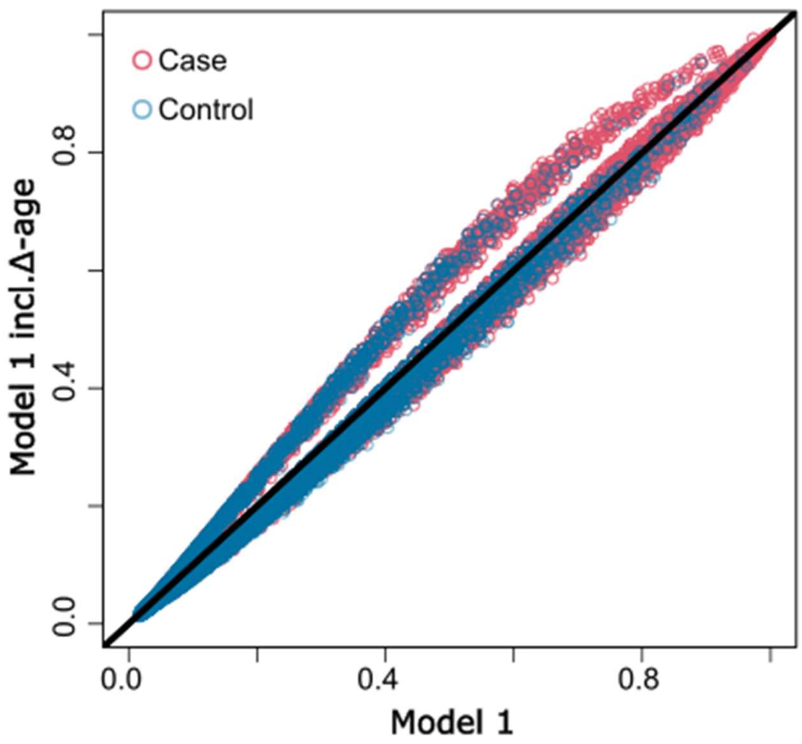

Figure S 2: Reclassification plot between Cox model 1 with and without including Δ-age at a 10-year time point for hazard estimation. The diagonal line represents the identity line. Red circles indicate cases, and blue circles represent controls. The inclusion of Δ-age shifts the classification, with cases being assigned higher risk and controls lower risk, as indicated by the slight upward deviation of red points and the downward shift of blue points relative to the diagonal. Model 1 includes age and gender.

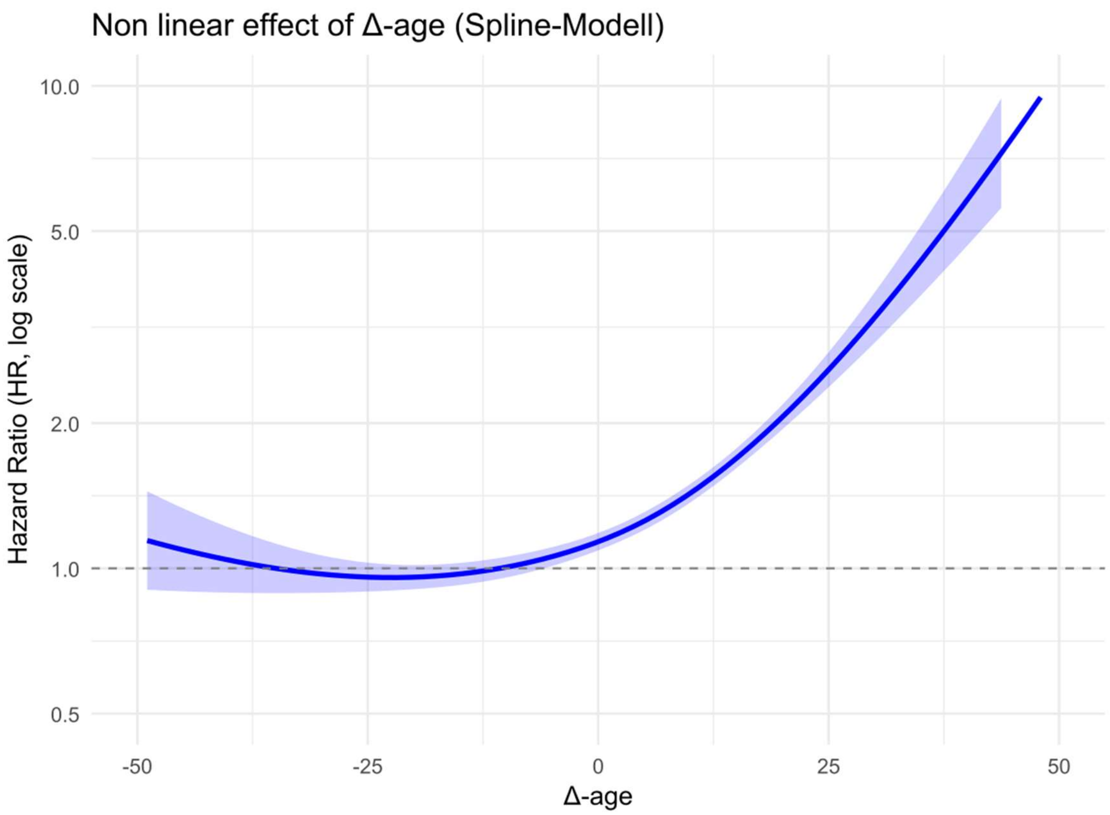

Figure S 3: Non-linear association between Δ-age and hazard ratio (HR) based on a Cox spline model. The x-axis represents the difference between AI-predicted ECG age and chronological age (Δ-age), and the y-axis shows the estimated hazard ratio on a logarithmic scale. The blue line depicts the spline-fitted HR, and the shaded area indicates the 95% confidence interval. The hazard increases steeply with higher Δ-age values, suggesting a pronounced non-linear risk increase for positive Δ-age. Model adjustments include age and gender.

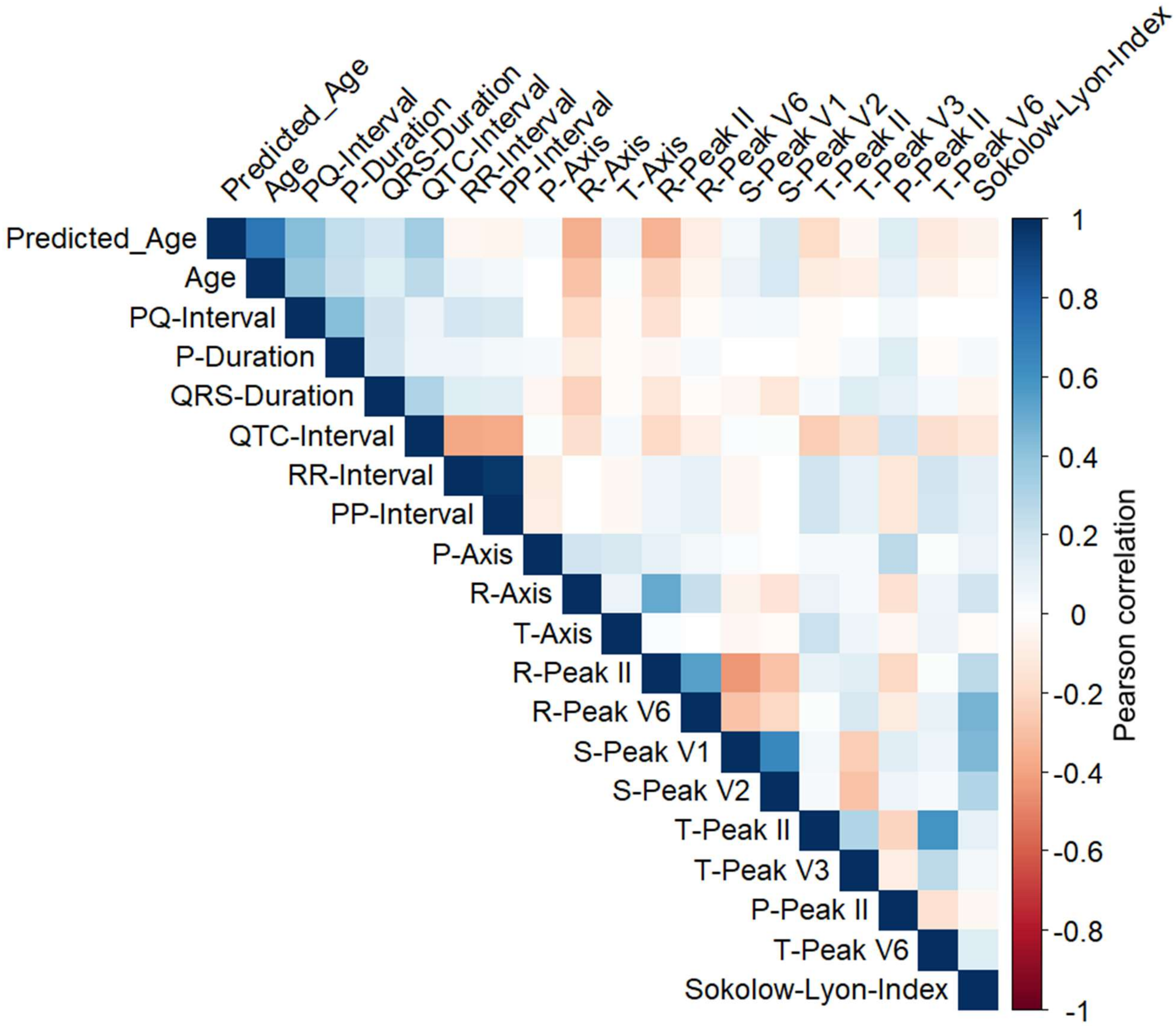

Figure S 4: Pearson correlation matrix of AI-ECG age, chronological age, and standard ECG parameters in the total cohort. The color intensity represents the strength and direction of the correlations, with blue indicating positive correlations and red indicating negative correlations. Darker colors indicate stronger correlations, while lighter colors represent weaker associations.

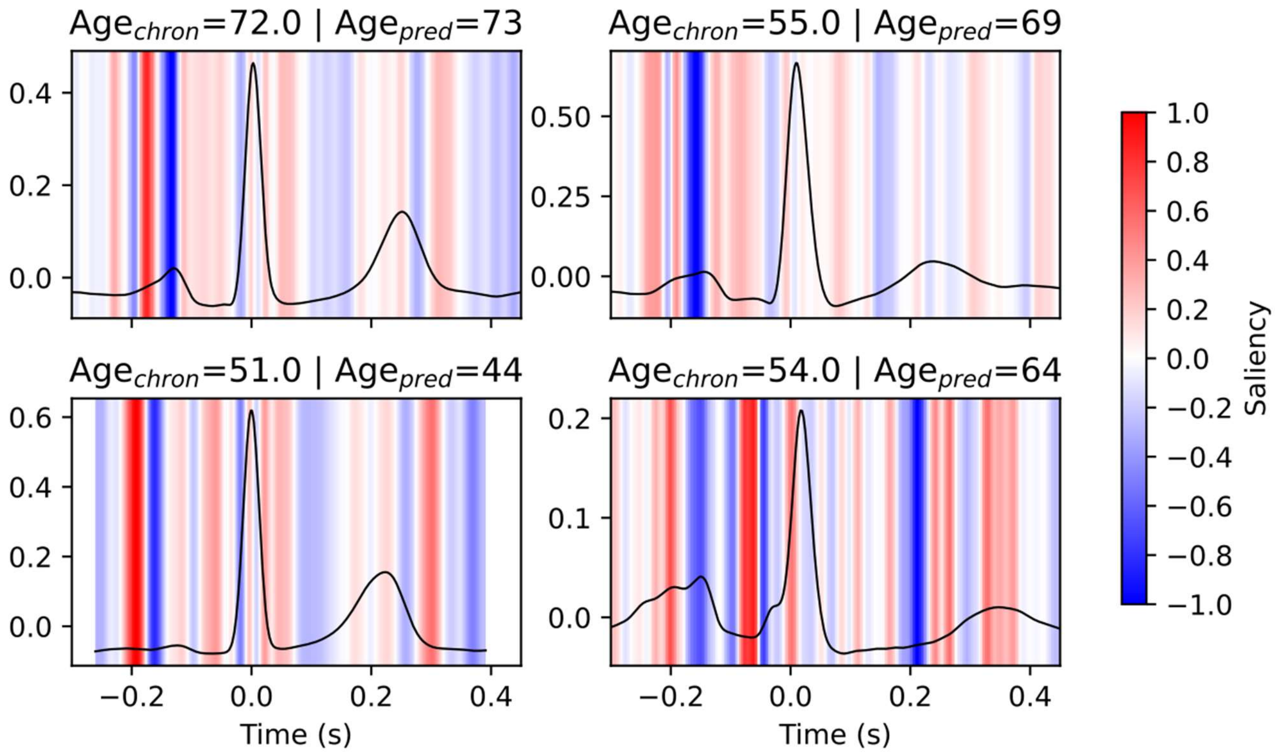

Figure S 5: Representative ECG beats and corresponding saliency maps for four patients. Each panel displays the median beat waveform (black line) for a single patient alongside the saliency map (color shading), with red indicating that an increase in voltage would increase the predicted age and blue indicating an increase in voltage would decrease predicted age. Notably, the regions surrounding the P wave and, in some cases, the T wave demonstrate high saliency values, suggesting that both atrial and ventricular features contribute to the model's estimation of biological age. The examples also illustrate inter-individual variation in saliency distribution, despite similar waveform morphology.

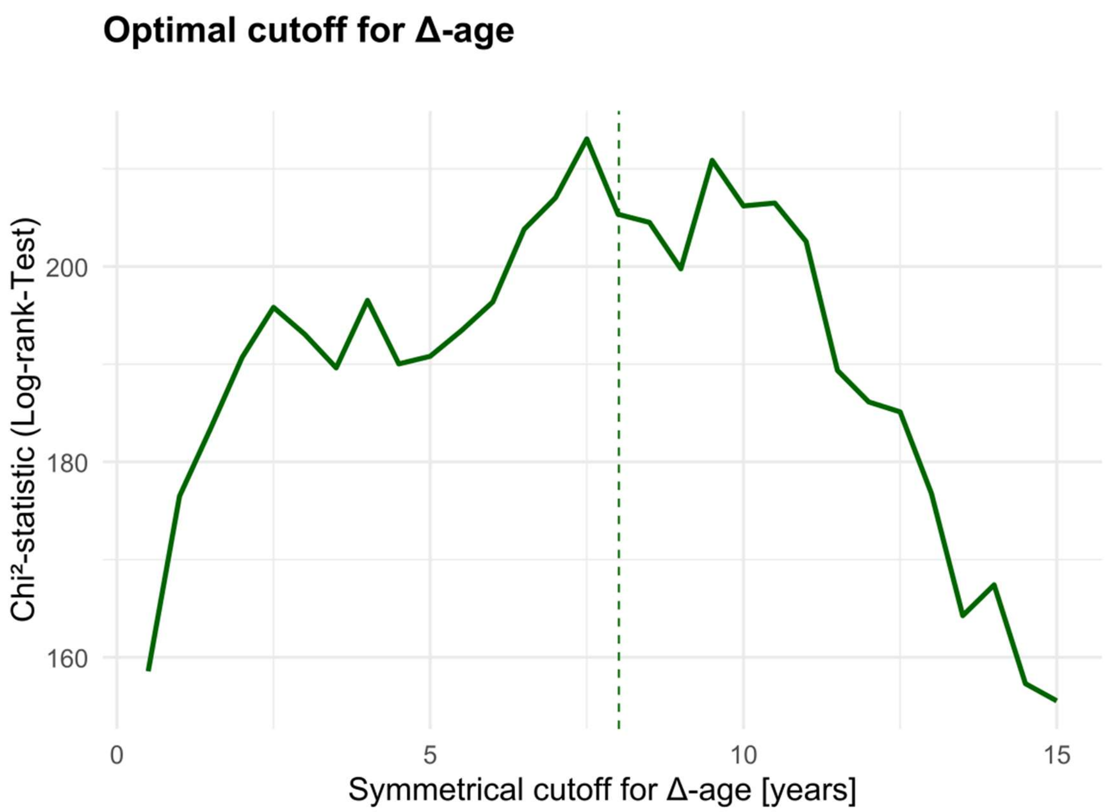

Figure S 6: Identification of the optimal Δ-age cutoff for survival stratification. The plot shows the Chi²-statistic (from the log-rank test) for symmetrical cutoff values of Δ-age, used to stratify the population into three groups (Δ-age < –cutoff, –cutoff ≤ Δ-age ≤ +cutoff, and Δ-age > +cutoff). The optimal cutoff, corresponding to the maximum Chi² value, was found at ±7.5 years, the dashed line at 8 years shows the cutoff we used in the analysis.
